# The HCF101 protein is an important component of the cytosolic iron–sulfur synthesis pathway in *Toxoplasma gondii*

**DOI:** 10.1371/journal.pbio.3003028

**Published:** 2025-02-06

**Authors:** Eléa A. Renaud, Ambre J. M. Maupin, Laurence Berry, Julie Bals, Yann Bordat, Vincent Demolombe, Valérie Rofidal, Florence Vignols, Sébastien Besteiro

**Affiliations:** 1 LPHI, Univ. Montpellier, CNRS, INSERM, Montpellier, France; 2 IPSiM, Univ. Montpellier, CNRS, INRAE, Institut Agro, Montpellier, France; University of Vermont Larner College of Medicine, UNITED STATES OF AMERICA

## Abstract

Several key cellular functions depend on proteins harboring an iron–sulfur (Fe-S) cofactor. As these Fe-S proteins localize to several subcellular compartments, they require a dedicated machinery for cofactor assembly. For instance, in plants and algae there are Fe-S cluster synthesis pathways localizing to the cytosol, but also present in the mitochondrion and in the chloroplast, 2 organelles of endosymbiotic origin. *Toxoplasma gondii* is a plastid-bearing parasitic protist responsible for a pathology affecting humans and other warm-blooded vertebrates. We have characterized the *Toxoplasma* homolog of HCF101, originally identified in plants as a protein transferring Fe-S clusters to photosystem I subunits in the chloroplast. Contrarily to plants, we have shown that HCF101 does not localize to the plastid in parasites, but instead is an important component of the cytosolic Fe-S assembly (CIA) pathway which is vital for *Toxoplasma*. While the CIA pathway is widely conserved in eukaryotes, it is the first time the involvement of HCF101 in this pan-eukaryotic machinery is established. Moreover, as this protein is essential for parasite viability and absent from its mammalian hosts, it constitutes a novel and promising potential drug target.

## Abstract 2

Iron-sulfur proteins require specialized assembly pathways and play vital roles in cellular functions. This study shows that the plastid-derived protein HCF101 functions in the cytosolic Fe-S assembly pathway in Toxoplasma, where it is crucial for Fe-S protein assembly and parasite viability.

## Introduction

Iron–sulfur (Fe-S) clusters are ancient inorganic cofactors of proteins which are essential in virtually all forms of life [1]. These cofactors are found in a variety of proteins involved in numerous electron transfer and metabolic reactions that support key housekeeping cellular functions like respiration, photosynthesis, DNA repair and replication, protein translation, RNA modifications, but also in regulatory proteins and environmental signals sensors [2,3]. The most common clusters, which in most Fe-S proteins function as electron transfer groups [4], are in the rhombic [2Fe-2S], cubane [4Fe-4S], or more rarely the intermediate [3Fe-4S] configuration. Assembly of these cofactors is a carefully controlled process in order to avoid cellular toxicity arising from the accumulation of free Fe and S, which are highly reactive. Different Fe-S cluster assembly pathways developed early in evolution to synthesize the clusters and can be found in archaea, bacteria, and then evolved to mitochondrial, plastidic and cytosolic Fe-S assembly machineries in eukaryotes [5,6]. Thus, eukaryotic Fe-S cluster assembly machineries found in organelles of endosymbiotic origin were inherited from Alphaproteobacteria (the “iron sulfur cluster,” or ISC, pathway in the mitochondrion) and Cyanobacteria (the “sulfur mobilization,” or SUF, pathway in plastids), while a specific machinery (“cytosolic iron–sulfur cluster assembly,” or CIA) is used for the biogenesis of cytosolic and nuclear Fe-S proteins.

While the individual components of these Fe-S assembly machineries display some structural diversity, they involve similar biochemical steps [7]. Typically, a cysteine desulfurase will generate S from L-cysteine, scaffold proteins, and chaperones will provide a molecular platform allowing the assembly of Fe and S into a cluster, and finally carrier proteins will deliver the cluster to target apoproteins. This happens in a similar fashion but with a specific molecular machinery in the 3 subcellular compartments in which Fe-S cluster synthesis takes place in eukaryotes, with a main difference that the early step of CIA depends on the mitochondrial machinery. In fact, although de novo cluster assembly has been suggested to happen in the cytosol of mammalian cells [8], experimental evidence in a large variety of eukaryotes points to the requirement of mitochondrial ISC components, in particular the Nfs1 cysteine desulfurase, to generate an S precursor that will be translocated to the cytosol [9–11]. In mammals and yeast, P-loop NTPases NUBP1 and NUBP2 are the scaffolds for initial [4Fe-4S] Fe-S cluster assembly in the CIA system [12] and electrons are provided by DRE2, together with NDOR1 (ATR3 in plants) [13]. Noticeably, some eukaryotes like plants lack a NUBP2 homolog and the scaffolding complex is instead composed of a dimer of NBP35, the NUBP1 homolog [14]. NAR1, a protein containing 2 [4Fe-4S] clusters, then acts as the Fe-S carrier and associates with the CIA targeting complex (CTC) [15]. The CTC, which will recognize client apoproteins through direct interactions and mediate the insertion of the Fe-S cluster, typically comprises MET18/MMS19 (plant/human nomenclature), CIA1/CIAO1, and AE7/CIAO2B [16].

*Toxoplasma gondii* is a widespread obligate intracellular parasitic protist belonging to the phylum Apicomplexa. Infection is usually harmless to immunocompetent individuals, but can lead to severe life-threatening disease in developing fetuses and immunocompromised individuals [17]. This parasite contains 2 organelles of endosymbiotic origin: a mitochondrion and a relict plastid called apicoplast, which are both of high metabolic importance [18]. Consequently, like in plants and algae *T*. *gondii* harbors 3 distinct Fe-S cluster synthesis pathways: the CIA in the cytosol, ISC in the mitochondrion, and SUF in the apicoplast [19–21]. The SUF pathway being essential for the survival of the parasite and absent from its mammalian host, its components and client proteins are particularly attractive as potential drug targets [22], which prompted us to look for Fe-S proteins absent from mammals but present in plant and apicomplexan parasites. Investigating the Fe-S cluster assembly components in protists, which are highly divergent phylogenetically from the canonical yeast or mammalian cell models, can bring interesting insights into the evolution of this molecular machinery [23,24].

Here, we report the characterization of the *T*. *gondii* homolog of the high-chlorophyll-fluorescence 101 (HCF101) protein, which is typically a plastid-associated Fe-S transfer protein in the plant *Arabidopsis thaliana* [25,26]. Our work shows that TgHCF101 is essential for parasite viability and we demonstrate for the first time its implication in the eukaryotic CIA pathway.

## Results

### TgHCF101 is a cytosolic protein

**Fig 1:**
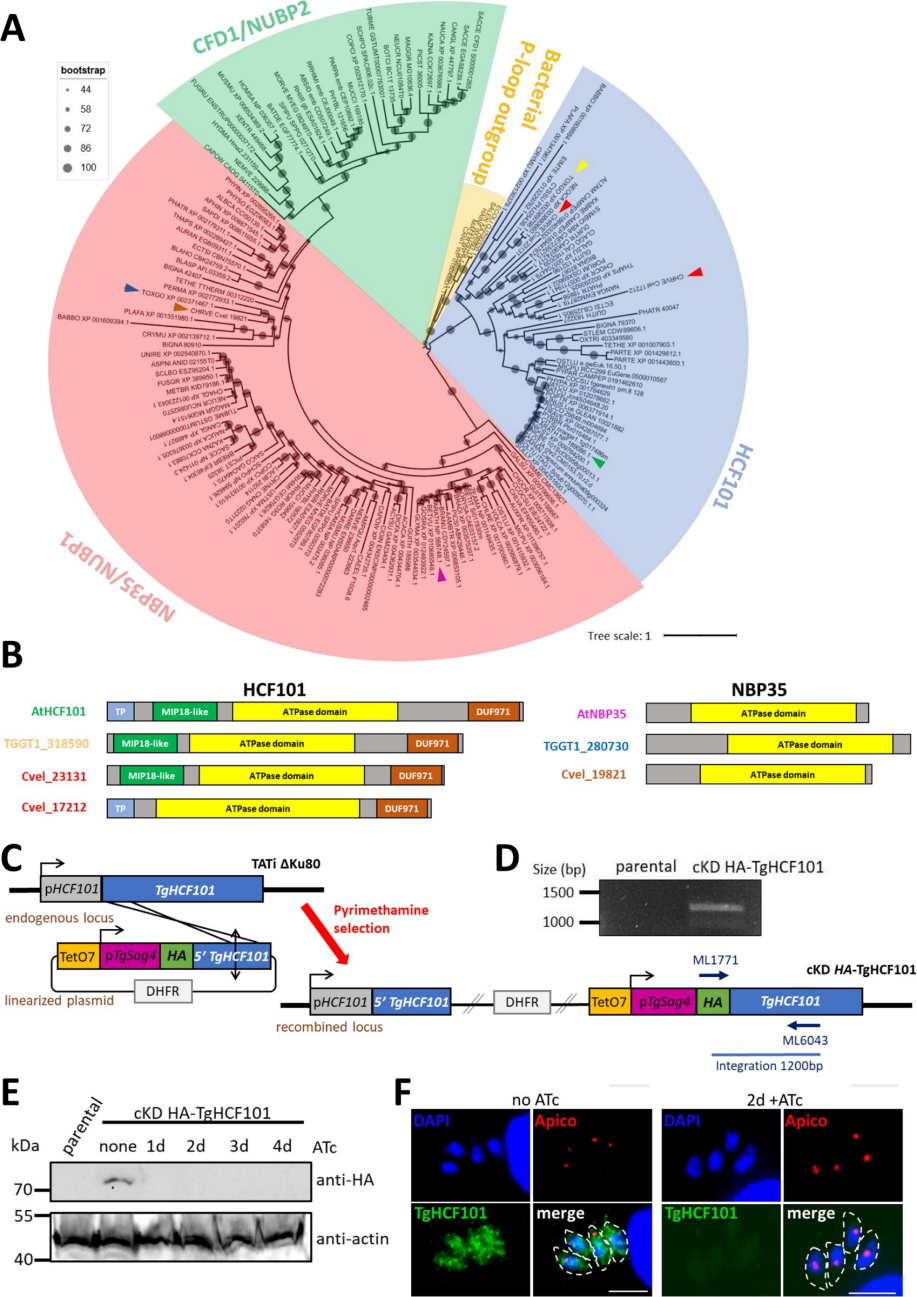
TgHCF101 is a cytosolic protein. ** (A)** Evolutionary relationship of proteins of the MRP family. Eukaryotic sequences from HCF101 homologs were aligned and submitted to phylogenetic analysis with the maximum likelihood method. Scale bar represents 1 residue substitution per site. Bacterial P-loop ATPase sequences were used as an outgroup. HCF101 from *A*. *thaliana* is indicated by a green arrowhead, TgHCF101 by a yellow arrowhead and the 2 homologs present in *C*. *velia* are indicated by red arrowheads. NBP35 homologs from *A*. *thaliana*, *T*. *gondii*, and *C*. *velia* are indicated by purple, blue, and brown arrowheads, respectively. **(B)** Schematic representation of homologs for HCF101 in *A*. *thaliana* (AtHCF101), in *T*. *gondii* (TGGT1_318590) and in *C*. *velia* (Cvel_23131, Cvel_17212), and homologs for NBP35 in *A*. *thaliana* (AtNBP35), in *T*. *gondii* (TGGT1_280730), and in *C*. *velia* (Cvel_19821). Main domains are highlighted on the sequences; TP: predicted transit peptide, MIP18-like domain (or Domain of unknown function 59, DUF59), the ATPase domain and DUF971. **(C)** Strategy for generating the inducible knockdown of *TgHCF101* by promoter replacement and simultaneous N-terminal tagging of the TgHCF101 protein in the TATi ΔKu80 cell line. **(D)** Diagnostic PCR for checking correct integration using the primers mentioned in (**C**), on genomic DNAs of a transgenic parasite clone and of the parental strain. **(E)** Immunoblot analysis of the cKD-TgHCF101 mutant and parental line showing efficient tagging and down-regulation of TgHCF101 starting at 24 h of treatment with ATc. Actin was used as a loading control. **(F)** Immunofluorescence assay showing a cytosolic signal for TgHCF101 protein (labeled with an anti-HA antibody), with no particular co-localization with the apicoplast (Apico, labeled with anti-PDH-E2 antibody), and total depletion of the protein after 48 h of ATc treatment. DNA was stained with 4′,6-diamidino-2-phenylindole (DAPI). Scale bar = 5 μm. The data underlying this figure can be found in S1 Table and in https://doi.org/10.6084/m9.figshare.28238195.

HCF101 is a putative P-loop containing nucleoside triphosphate hydrolase that belongs to the Mrp/NBP35 family (also called ApbC) of dimeric iron–sulfur carrier proteins that are found ubiquitously in all domains of life and thus likely appeared early during evolution [27,28]. Compared with NBP35, in addition to the nucleotide hydrolase domain HCF101 also contains a N-terminal DUF59/MIP18-family domain [29], as well as a C-terminal domain of unknown function (DUF971). We performed homology searches in the database to retrieve members of the Mrp/NPB35 family encoded by the *T*. *gondii* genome. Besides TgNBP35 that has an unusual mitochondrial localization in *T*. *gondii* [20,24], in line with previous studies [24,30], we identified TGGT1_318590 as a potential homolog of HCF101. A broader phylogenetic analysis that included members of the Mrp/NPB35 family from a wide variety of eukaryotes (S1 Table), revealed that this putative *T*. *gondii* homolog clearly segregates with members of the HCF101 group, clearly distinct from NBP35 homologs (Fig 1A). Moreover, its primary sequence analysis confirmed a 3 domain-organization typical of HCF101 proteins, while NBP35 homologs mostly consist of the central ATPase domain (Fig 1B). Of note, our analysis, in accordance with a previous study [24], confirmed the absence of a CFD1 homolog in Apicomplexa, contrarily to what has been suggested by a recent report [31]. In conclusion, TGGT1_318590 may be named TgHCF101.

In *A*. *thaliana*, the *HCF101* mutant was found to be impaired in photosynthesis [32]: interfering with HCF101 function has an impact on the maturation of both photosystem I (PSI) and ferredoxin-thioredoxin reductases, which contain [4Fe-4S] clusters [25,26]. The apicoplast harbored by apicomplexan parasites has lost the ability to perform photosynthesis [33], and some members of the Apicomplexa phylum, like *Cryptosporidium*, have even completely lost the plastid [34] while it has retained a HCF101 homolog (Figs 1A and S1). Thus, a function linked to photosystem seems unlikely for apicomplexan parasites. Sequence analysis confirmed that, in contrast to plant homologs, TgHCF101 lacks a predicted N-terminal transit peptide, which is typically needed for plastid import (Fig 1B). Interestingly, *Chromera velia*, a close non-parasitic relative of Apicomplexa whose plastid has retained its photosynthetic capacity [35], has 2 HCF101 isoforms: one with a predicted transit peptide (thus potentially addressed to the plastid), and one without (Figs 1 and S1). Specific analysis of selected eukaryotic HCF101 homologs showed that Apicomplexa proteins, like ciliates and dinoflagellates, form distinct groups that are clearly separate from the Archaeplastida group of plants, as well as algae bearing plastid-associated HCF101 isoforms (S1 Fig). Interestingly, while the transit peptide-bearing *C*. *velia* isoform segregates with homologs of plastid-containing eukaryotes, the second isoform is closer to the Apicomplexa homologs (S1 Fig). A recent study suggests that the plastid-located HCF101 likely evolved from an ancestral cytosolic version of the protein [24]. This led us to hypothesize that apicomplexan HCF101 would be involved in a cytosolic-related, rather than in a plastid-associated, Fe-S cluster transport function.

To assess experimentally the localization of TgHCF101, we first tagged an ectopic copy with a C-terminal GFP and observed a cytosolic localization (S2A Fig), in line with what had been described previously when an additional copy of TgHCF101 bearing a C-terminal Human influenza hemagglutinin (HA) tag was expressed [24]. However, a stable TgHCF101-GFP cell line could not be established, suggesting the potential importance of C-terminal accessibility for proper protein function. We then next generated a transgenic cell line in which we modified the 5′ region of the *TgHCF101* gene by homologous recombination to replace the endogenous promoter by an inducible-Tet07*SAG4* promoter, and at the same time adding a sequence coding for a N-terminal HA tag (Fig 1C and 1D). In this conditional knock-down (cKD) HA-TgHCF101 cell line, the addition of anhydrotetracycline (ATc) can repress transcription of *TgHCF101* through a Tet-Off system [36]. Two independent transgenic clones were obtained and found to behave similarly in the initial phenotypic assays we performed, so only one will be described in details here.

Immunoblot analysis showed an N-terminal HA-tagged protein at around 70 kDa (the predicted molecular mass for TgHCF101) and depletion of the protein was efficient upon addition of ATc (Fig 1E). Immunofluorescence assay (IFA) showed a punctate cytosolic signal for TgHCF101, clearly distinct from the apicoplast, and efficiently depleted by the addition of ATc (Fig 1F). Disruption of the apicoplast-located SUF pathway has a strong impact on the lipoylation of the E2 subunit of the pyruvate dehydrogenase (PDH-E2) by Fe-S-containing lipoate synthase LipA [19,22]. However, depletion of TgHCF101 did not seem to have an impact on the lipoylation profile of PDH-E2 (S2B Fig). Disruption of the SUF pathway also typically impacts the apicoplast-located isoprenoid synthesis, which involves 2 Fe-S oxidoreductases (IspG and IspH) and has been shown to have downstream consequences on the glycosylation of proteins involved in parasite motility [22]. We thus assessed the gliding motility of the parasites after TgHCF101 depletion and found that it was only affected after 4 days of ATc treatment, which might be reflecting long-term impact of TgHCF101 depletion on parasite fitness rather than a direct consequence of apicoplast-related Fe-S cluster synthesis (S3A and S3B Fig).

Altogether, our results indicate that TgHCF101 is not associated with the apicoplast-located SUF pathway, but localizes to the cytosol instead.

### TgHCF101 is essential for parasite growth

**Fig 2:**
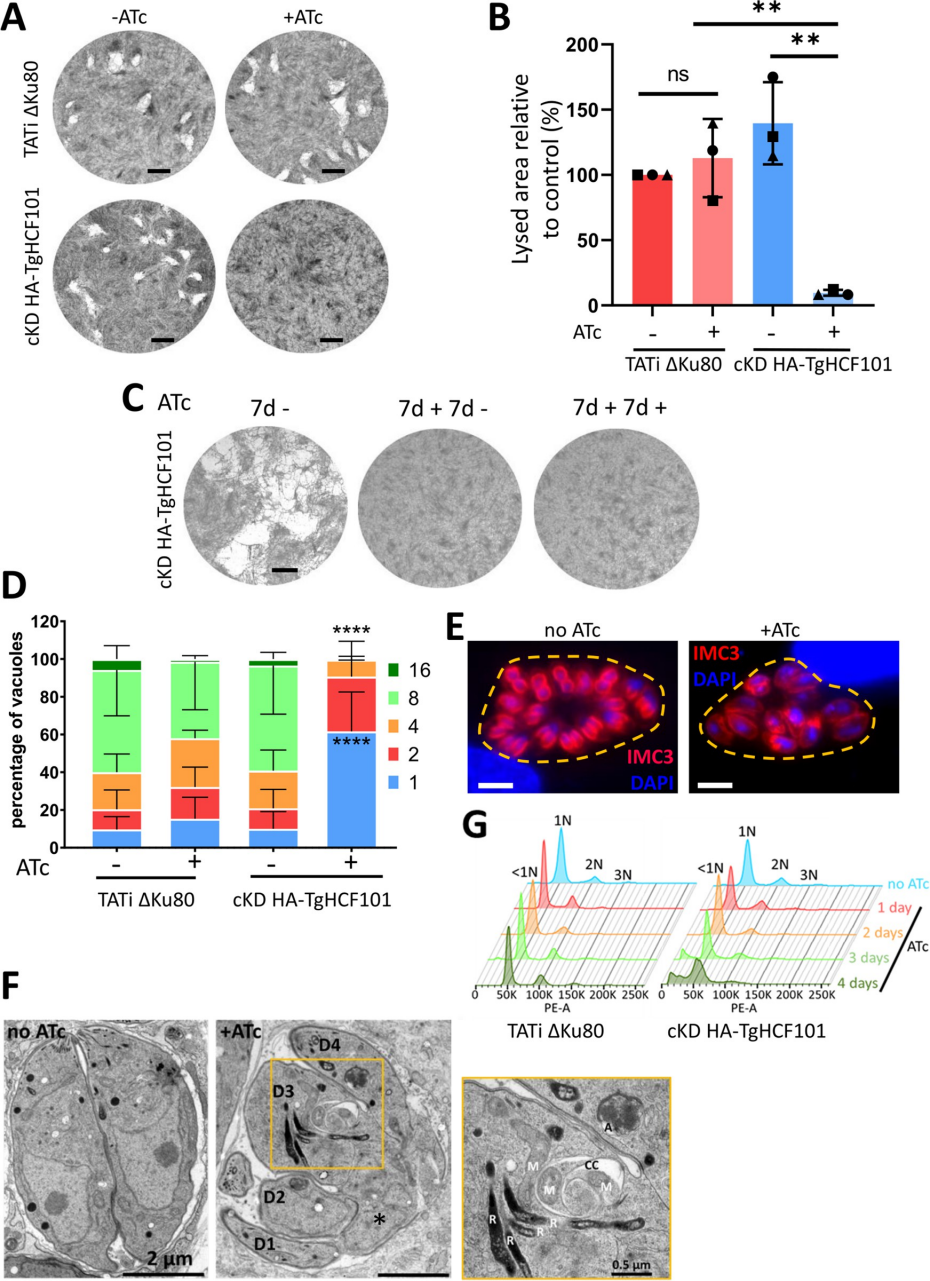
TgHCF101 is essential for parasite growth and survival. **(A)** Plaque assays were carried out by infecting a monolayer of HFFs with TATi ΔKu80 or cKD-TgHCF101 cell lines for 7 days in the presence or absence of ATc. Scale bar = 2 mm. **(B)** Quantification of plaque area observed in (A). Results are expressed as percentage of lysed area relative to control (TATi ΔKu80 -ATc, set as 100% for reference). Values represented are mean ± SD from *n* = 3 independent biological replicates, ** *p*-value ≤0.01, Student *t* test. **(C)** Plaque assays were carried out with cKD-TgHCF101 parasites as described in (A) but for the ‘7d+7d-‘ condition, ATc was washed-out after 7 days and parasites were allowed to grow for another 7 days without ATc, while in the ‘7d+7d+’ condition, ATc treatment was maintained for 7 more days. The ‘7d –‘control was kept without ATc for 7 days of growth. Scale bar = 2mm. **(D)** Replication assay of parental (TATi ΔKu80) and transgenic cell lines (cKD-TgHCF101): parasites were pre-cultured for 48 h in the presence or absence of ATc and allowed to invade HFF-coated coverslips for another 24 h in the presence or absence of ATc, for a total of up to 72 h of treatment with ATc. Number of parasites per vacuole was quantified for each condition and expressed as a percentage, 200 vacuoles were counted for each condition. Values represented are mean ± SD of 3 independent biological replicates, **** *p*-value ≤0.0001 by two-way ANOVA with Dunnett’s multiple comparison test, showing a significant difference when comparing the TATi ΔKu80 control and cKD-TgHCF101+ATc parasites for percentage of vacuoles containing 1 or 8 parasites. **(E)** Immunofluorescence assay of cKD-TgHCF101 parasites showing asynchronous division of parasites growing in the same vacuole (outlined with a yellow dotted line) upon TgHCF101 depletion (+ATc condition: parasites pre-incubated for 48 h with ATc and allowed to invade coverslips for another 48 h in the presence of ATc). Individual parasites and budding daughter cells are outlined by anti-IMC3 antibody staining (magenta), DNA was stained with DAPI. Scale bar = 5 μm. **(F)** Electron microscopy of cKD-TgHCF101 parasites pre-incubated with ATc for 24 h before being released from their host cell and allowed to reinvade for 24 h in the presence (+ATc) of ATc, or grown in absence of ATc (-ATc). Inset shows magnification of a selected region. D: daughter bud, R: rhoptry, A: apicoplast, M: mitochondrion, CC: cytoplasmic cleft. The asterisk denotes unincorporated nuclear material. Scale bar = 2 μm (0.5 μm for inset magnification). **(G)** DNA content analysis by flow cytometry on TATi ΔKu80 and cKD-TgHCF101 parasites treated or not with ATc up to 4 days and stained with propidium iodide. 1N, 2N, 3N represent the ploidy, with <1N corresponding to parasites with less than 1 full nuclear DNA content. The data underlying this figure can be found in S1 Data and http://flowrepository.org/id/FR-FCM-Z8SR.

We next assessed the impact of TgHCF101 depletion on the parasite’s lytic cycle: We evaluated the capacity of TgHCF101-depleted tachyzoites (a highly replicative and highly invasive form responsible for the acute form of the disease caused by *T*. *gondii*) to generate lysis plaques. For this, we infected a monolayer of host cells in absence or continuous presence of ATc for 7 days (Fig 2A and 2B). Depletion of TgHCF101 completely prevented plaque formation. While absence of plaques highlights a fitness problem for the parasites, it does not necessarily implies their death: for instance, in response to stress, tachyzoites can convert into a slow-growing and cyst-enclosed persisting stage called bradyzoites (associated with the chronic form of the disease), that can reactivate when they encounter more favorable conditions [37]. We thus performed a similar experiment, in which the ATc was washed out at the end of the 7-day incubation, and then incubated the parasites for an extra 7 days in the absence or presence of ATc before evaluating plaque formation (Fig 2C). We did not observe any plaques after ATc removal, suggesting that the parasites were not able to recover after 7 days of TgHCF101 depletion, likely because they were dead.

To assess whether this defect in the lytic cycle is due to a replication problem, cKD HA-TgHCF101 parasites were preincubated in ATc for 48 h and then released mechanically, before infecting new host cells for an additional 24 h in ATc prior to parasite counting. We noted that incubation with ATc led to an accumulation of vacuoles with fewer parasites (Fig 2D). To get more precise insights into the impact of TgHCF101 depletion on parasite division, we labeled dividing parasites with inner membrane complex (IMC) protein IMC3 [38] to detect growing daughter cells (Fig 2E). *T*. *gondii* tachyzoites develop inside a mother cell by a process called endodyogeny [39] and this division is usually highly synchronous within the same parasitophorous vacuole, yet after 2 days of ATc treatment vacuoles showed a marked lack of synchronicity for daughter cell budding (Fig 2E). Investigation of the consequences of TgHCF101 depletion on developing parasites by electron microscopy highlighted important defects on cytokinesis, including incomplete daughter cell budding or defaults in organellar segregation (Figs 2F and S4). We also observed occasional vacuoles and cytoplasmic clefts (Figs 2F and S4). However, the overall appearance of organelles seemed essentially normal (Figs 2F and S4). To get a more general overview, we performed IFA using organelle-specific markers, which confirmed that TgHCF101 depletion leads to defaults in the replication or segregation of organelles, some daughter cells appearing devoid of apicoplast, mitochondrial or nuclear material (S5 Fig). Finally, through propidium iodide labeling and flow cytometer-based analysis of DNA content, we observed that long-term depletion of TgHCF101 led to the emergence of an increasing subpopulation of parasites with sub-1N DNA content, thus smaller than the typical haploid 1N DNA content of the parasites (Fig 2G), suggesting an impairment in DNA synthesis.

Overall, our results indicate that TgHCF101 depletion leads to important and, apparently, irreversible defects in parasite replication and growth.

### Depletion of TgHCF101 induces stress response mechanisms

**Fig 3:**
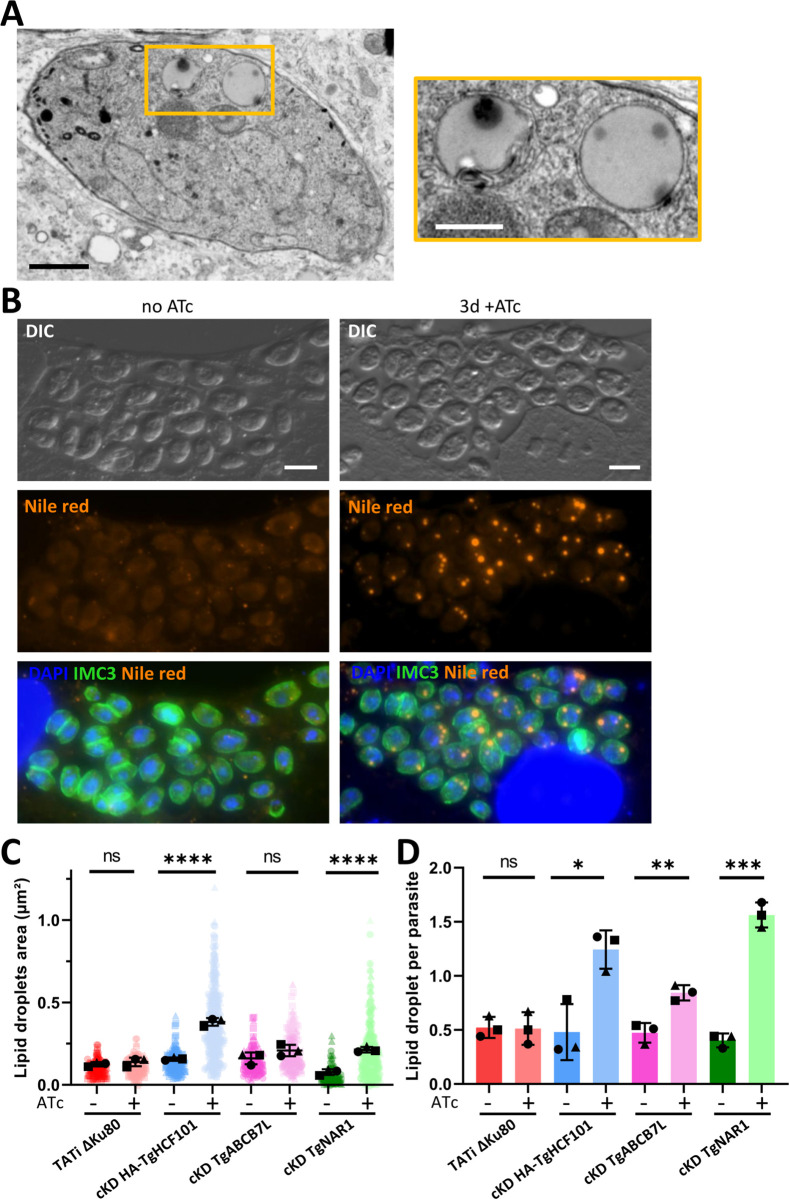
TgHCF101 depletion induces lipid droplet accumulation. **(A)** Electron microscopy of cKD-TgHCF101 parasites pre-incubated with ATc for 48 h and allowed to reinvade for 24 h in the presence of ATc. Scale bar = 1 μm. The panel on the right corresponds to a magnification of the selection on the left panel, highlighting lipid droplets. Scale bar = 500 nm. **(B)** Fluorescent imaging of parasites from the cKD-TgHCF101 cell line treated for 72 h with ATc or in grown the absence of ATc, shows an accumulation of lipid droplets upon TgHCF101 depletion. LDs were detected with Nile red (orange), parasites are outlined with an anti-IMC3 antibody (green) and DNA is stained with DAPI. DIC = Differential interference contrast. Scale bar = 5 μm. **(C, D)** Correspond to the quantification of lipid droplet area and number, respectively, and 100 parasites were analyzed per condition. The parental (TATi ΔKu80) and transgenic parasites (cKD HA-TgHCF101, cKD TgABCB7, cKD TgNAR1) were grown in absence of ATc, or in presence of ATc for 72 h. Values are represented as the mean ± SD of *n* = 3 independent biological replicates (different symbols represent different series); ns, not significant (*p*-value >0.05), * *p*-value ≤0.05, ** *p*-value ≤0.01, *** *p*-value ≤0.001, **** *p*-value ≤0.0001, Student’s *t* test. The data underlying this figure can be found in S1 Data. cKD, conditional knock-down; LD, lipid droplet; SD, standard deviation.

Through electron microscopy analysis, we also noticed that the depletion of TgHCF101 induced the appearance of structures resembling lipid droplets (LDs) in several parasites (Figs 3A and S4). In order to properly quantify this, we used Nile red, a selective fluorescent *stain* for neutral lipids, and microscopic imaging (Fig 3B). We noticed a strong increase in both the number and size of LD in cKD HA-TgHCF101 parasites incubated for 3 days with ATc (Fig 3C and 3D). To see if this was a common feature induced by abolishing Fe-S synthesis, we quantified LDs in mutants of the mitochondrial (TgISU1) and apicoplast (TgSUFC) pathways [19]. Depletion of these proteins did not lead to an increase in LD formation (S6A and S6B Fig), indicating that LD induction may be linked to a pathway distinct from the mitochondrial or plastid-related Fe-S cluster assembly machinery. We then also tested a recently characterized mutant for TgABCB7L (a mitochondrial transporter of a sulfur precursor upstream of the CIA pathway [40]) and a mutant of the CIA shuttle protein TgNAR1 (www.ToxoDB.org entry TGGT1_242580) that we generated, and which is important for parasite fitness (S7 Fig). Interestingly, both these CIA-related mutants induced an increase in LD numbers (although more modest for the TgABCB7L mutant), while only the TgNAR1 led to a statistically significant increase in LD size (Fig 3C and 3D).

**Fig 4:**
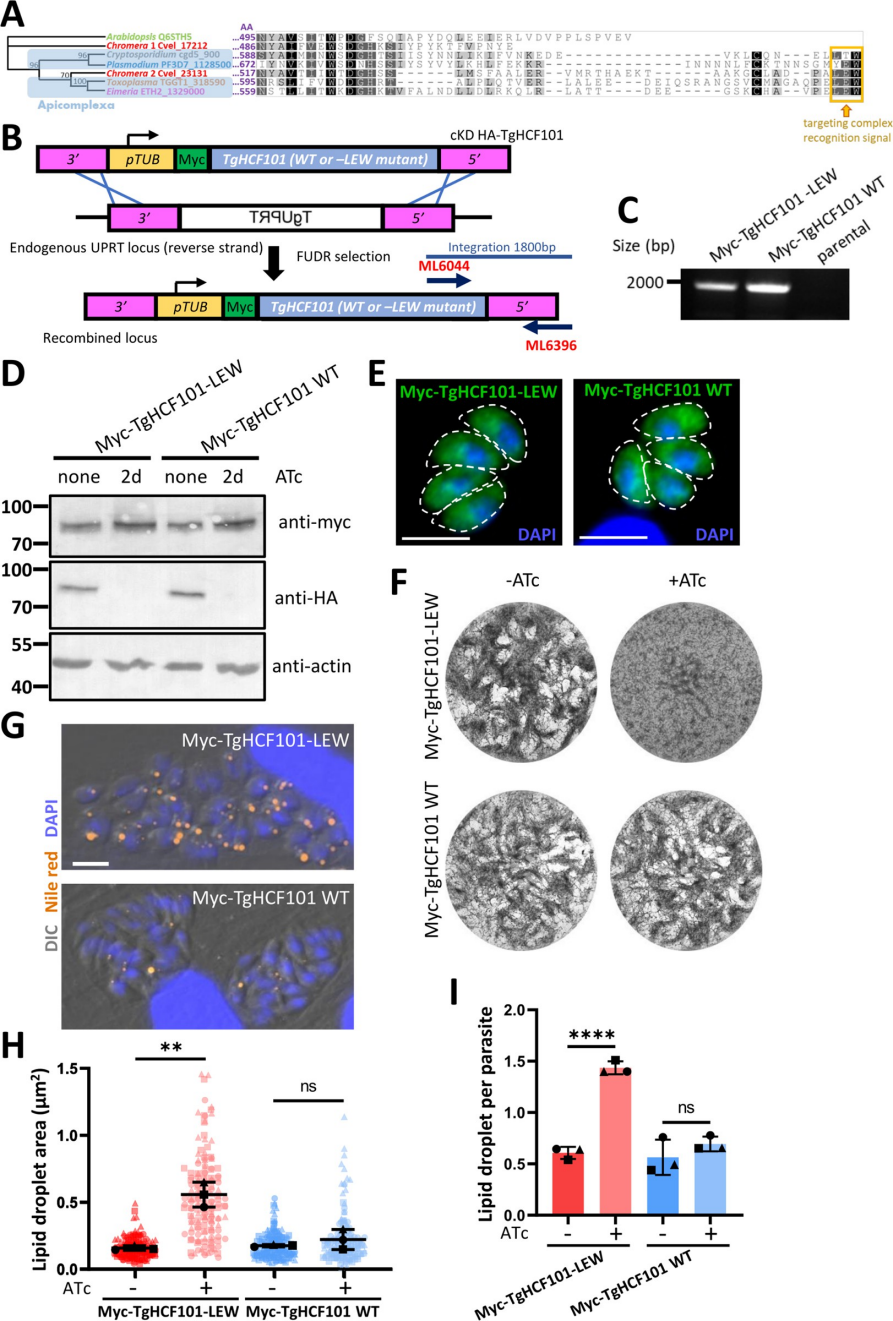
A C-terminal TCR-like motif is necessary for TgHCF101 function. **(A)** Alignment of the C-terminal region of HCF101 homologs from different eukaryotes, including the plant *A*. *thaliana*, the 2 isoforms of Apicomplexa-relative photosynthetic algae *C*. *velia*, as well as several Apicomplexa species (*T*. *gondii*, *Eimeria tenella*, *P*. *falciparum*, and apicoplast-less *Cryptosporidium parvum*). The tryptophan-containing TCR signal is highlighted in yellow. Consensus tree was obtained with protein alignment and bootstrap values (500 replicates) are indicated at the base of the nodes. **(B)** Schematic representation of the strategy for generating cell lines expressing myc-tagged wild-type (WT) or TCR motif-deleted (-LEW) copies of TgHCF101 by integrating an extra copy of the gene of interest by double homologous recombination at the *Uracil Phosphoribosyltransferase* (*UPRT*) locus. Negative selection with 5-fluorodeoxyuridine (FUDR) was used to select transgenic parasites based on their absence of UPRT expression. **(C)** Diagnostic PCR for verifying integration at the *UPRT* locus thanks to the primers described in **B. (D)** Immunoblot analysis showing expression of the additional myc-tagged copies upon depletion of the ATc-regulated HA-tagged copy. **(E)** Immunofluorescence with anti-myc antibody confirms the cytoplasmic localization of the extra copies. Parasite shape is outlined. DNA was stained with DAPI. Scale bar = 5 μm. **(F)** Plaque assay showing restoration of growth by the additional WT copy upon depletion of the ATc-regulated copy, contrarily to the TCR mutant. **(G)** Representative images of vacuoles (outlined) containing parasites grown continuously in the presence of ATc for 72 h and stained by Nile red (orange) for lipid droplets. DIC: differential interference contrast. DNA was stained with DAPI. Scale bar = 10 μm. **(H, I)** Correspond to the quantification of lipid droplet area and number, respectively; 100 parasites were analyzed per condition. WT or TCR-depleted (-LEW) parasites were grown in presence of ATc for 72 h. Values are represented as the mean ± SD of *n* = 3 independent biological replicates (different symbols represent different series); ns, not significant (*p*-value >0.05), ** *p*-value ≤0.01, **** *p*-value ≤0.0001, Student’s *t* test. The data underlying this figure can be found in S1 Data. HA, hemagglutinin; SD, standard deviation; TCR, targeting complex recognition.

As LD droplet induction seems to be a feature common to CIA pathway mutants and to the TgHCF101 mutant, we thought that TgHCF101 may play a role in this pathway. Additional supporting evidence comes from the characterization of a C-terminal targeting complex recognition (TCR) signal ending with an aromatic residue ([LIM]-[DES]-[WF]) in the clients proteins of the cytosolic Fe-S cluster machinery or their adaptors [41], which can be clearly identified in the apicomplexan HCF101 homologs (Fig 4A). Of note, this motif is also present in the *C*. *velia* isoform that has no transit peptide and thus is likely to have a plastid-independent function (Fig 4A). Importantly, the last 3 amino acids of TgHCF101 (LEW, Fig 4A) constitute a canonical TCR motif. Thus, from the cKD HA-TgHCF101 mutant, we generated cell lines in which we expressed either a myc-tagged wild-type copy or one deleted for this putative TCR signal (Fig 4B and 4C). As expected, expression of these additional copies was unaltered by ATc treatment (Fig 4D), and the corresponding proteins localized to the cytoplasm (Fig 4E). Upon depletion of the ATc-regulated copy, expressing the extra wild-type copy was efficient in restoring parasite growth (Fig 4F and 4G) and preventing LD accumulation (Fig 4H and 4I). Yet, in sharp contrast, expressing the TCR-deleted extra copy did not improve parasite fitness (Fig 4F and 4G) and did not prevent induction of LD formation (Fig 4H and 4I).

To assess if the induction of LD formation could play a role in the demise of the parasites, we used T863, a pharmaceutical inhibitor of the acyl coenzyme A:diacylglycerol acyltransferase 1 (DGAT1) enzyme, which is important for storing neutral lipids in cytoplasmic LD [42]. While treatment with T863 efficiently decreased the number of LDs (S6C, S6D, and S6E Fig), long-term incubation during plaque assays did not allow any recovery in parasite fitness (S6F Fig). This suggests that LDs are not detrimental to TgHCF101-depleted parasites (or at least not solely responsible for their demise), but instead could be part of an integrated stress response, as they have been shown in other eukaryotes to be up-regulated in response to cellular injuries including nutrient-related and oxidative stresses [43].

**Fig 5:**
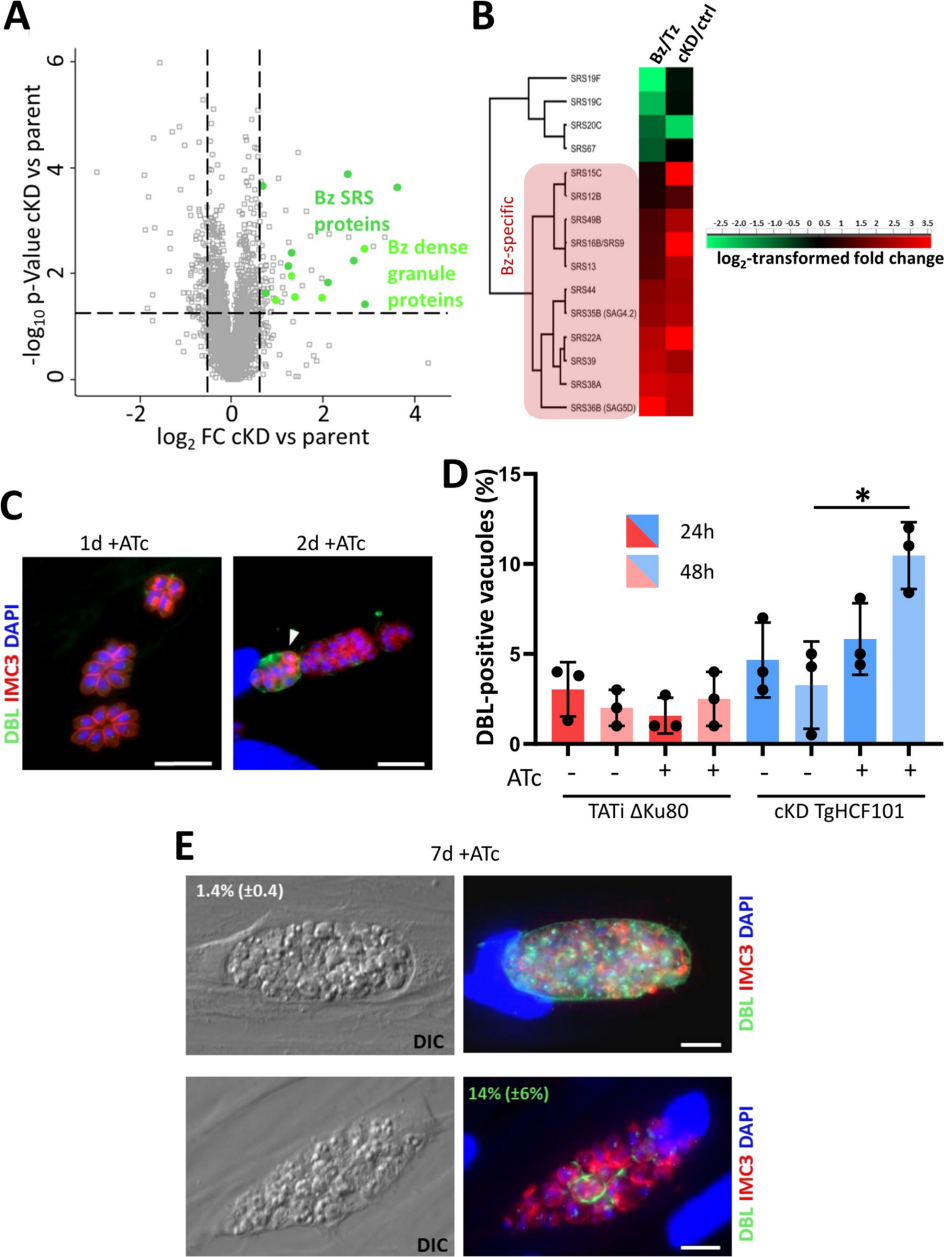
TgHCF101-depleted parasites express bradyzoite-specific markers but are unable to complete their conversion. **(A)** Volcano plot showing differential expression of proteins impacted by TgHCF101 depletion after 72 h of ATc treatment analyzed by label-free quantitative proteomic. X-axis corresponds to the log_2_ of the fold-change (FC) and the Y-axis corresponds to the -log_10_ of the *p*-value, when comparing cKD-TgHCF101 expression values to the TATi ΔKu80 parental cell line. Statistical analyses were performed with ANOVA on 4 independent biological replicates. Cut-offs were set at ≤1.5- or ≥1.5-FC and *p*-value ≤0.05. Significant hits corresponding to stage-specific protein are highlighted in green on the graph. **(B)** Clustering of bradyzoite (Bz) or tachyzoite (Tz)-specific proteins of the SRS family shows specific enrichment of bradyzoite proteins upon TgHCF101 depletion. **(C)** Immunofluorescence assay of cKD-TgHCF101 treated for 24 h or 48 h with ATc, cyst wall is labeled with DBL, parasites periphery is outlined with anti-IMC3 antibody and DNA is stained with DAPI. Scale bar = 10μm. **(D)** Corresponds to the quantification of the percentage of vacuoles presenting a DBL positive signal as shown in (**C**). Values are represented as the mean ± SD of *n* = 3 independent biological replicates, * *p*-value ≤0.05, Student’s *t* test. **(E)** Immunofluorescence assay of cKD-TgHCF101 treated for 7 days in the presence of ATc, cyst wall is labeled with DBL, parasites are outlined with anti-IMC3 antibody and DNA is stained with DAPI. The percentage of DBL positive vacuoles of corresponding size (more than 2 parasites per vacuole, top; or 2 parasites per vacuole or less, bottom) is specified as mean ± SD of *n* = 3 independent biological replicates. DIC = Differential interference contrast. Scale bar = 10 μm. The data underlying this figure can be found in S2 and S3 Tables and in S1 Data. cKD, conditional knock-down; SD, standard deviation.

Disrupting proteins involved in Fe-S cluster assembly may have a direct effect on the stability and expression levels of local Fe-S proteins. Thus, to get insights into the effect of TgHCF101 depletion at the molecular level, we performed global label-free quantitative proteomic analyses. Of course, this may also affect downstream cellular pathways or functions, and other pathways may also be up-regulated in compensation. cKD HA-TgHCF101 or TATi ΔKu80 parental control parasites were treated for 3 days with ATc prior to a global proteomic analysis and compared for protein expression. We selected candidates with a log_2_(fold change) ≤-0.55 or ≥0.55 (corresponding to a ~1.47-fold change in decreased or increased expression) and a *p*-value <0.05 (ANOVA, *n* = 4 biological replicates) and we completed this data set by selecting some candidates that were consistently and specifically absent from the mutant cell lines or only expressed in these (S2 and S3 Tables). Many proteins with higher abundance were bradyzoite stage-specific and more particularly components of the cyst wall or their cell surface [44,45] (Fig 5A and 5B and S2 Table). Tachyzoites can convert to the persistent bradyzoite form upon stress, so to verify if TgHCF101 depletion was inducing stage conversion we used a lectin from the plant *Dolichos biflorus*, which recognizes the SRS44/CST1 cyst wall glycoprotein in differentiating cysts [46] (Fig 5C and 5D). We could see that TgHCF101 depletion induced the appearance of up to 10% of lectin-labeled vacuoles during the first 48 h of intracellular growth in the presence of ATc. However, when parasites were kept for up to a week in the presence of ATc, the proportion of lectin-positive vacuoles plateaued at 14%, and only a very limited number of vacuoles looked like bona fide mature cysts (less than 2% were both lectin-positive and containing more than 2 parasites) (Fig 5E).

These data suggest that depletion of TgHCF101 leads to a cellular stress that initiates stage conversion into the bradyzoite stage, but that the differentiation process into mature cysts cannot be completed.

### Depletion of TgHCF101 affects specifically cytosolic and nuclear Fe-S proteins

The label-free quantitative proteomic analyses also highlighted proteins which were less expressed in absence of TgHCF101. Among them were a surprisingly large number of rhoptry bulb proteins (S3 Table and S8A Fig). Rhoptries are club-shaped organelles that comprise a narrow tubular neck opening at the anterior pole of the parasite, and a most posterior bulbous part; the proteins secreted from these different sub-compartments are either involved in invasion and parasitophorous vacuole formation, or in the modulation of host cell defenses, respectively [47]. We assessed if there was any major impact of TgHCF101 depletion on rhoptry morphology or function. We performed IFA with anti-armadillo repeats only protein (TgARO), a protein homogenously anchored to the surface of the rhoptry membrane [48], and found no particular defect in rhoptry morphology or positioning (S8B Fig), in accordance with our initial electron microscopy analysis (Fig 2F). Quantification of intracellular evacuoles (rhoptry-secreted vesicular clusters) did not indicate any particular problem in the secretory capacity of the organelles upon TgHCF101 depletion (S8C Fig). Finally, we assessed by immunoblot the expression profile of one of the potentially less-expressed rhoptry bulb protein but found only a slight alteration in the expression profile (a modest increase in the non-mature from of the protein), but not in the overall amount of protein (S8D Fig). We conclude that although TgHCF101 depletion may lead to a secondary impact on rhoptry content, it does not extensively impact organelle morphology or secretory function.

**Fig 6:**
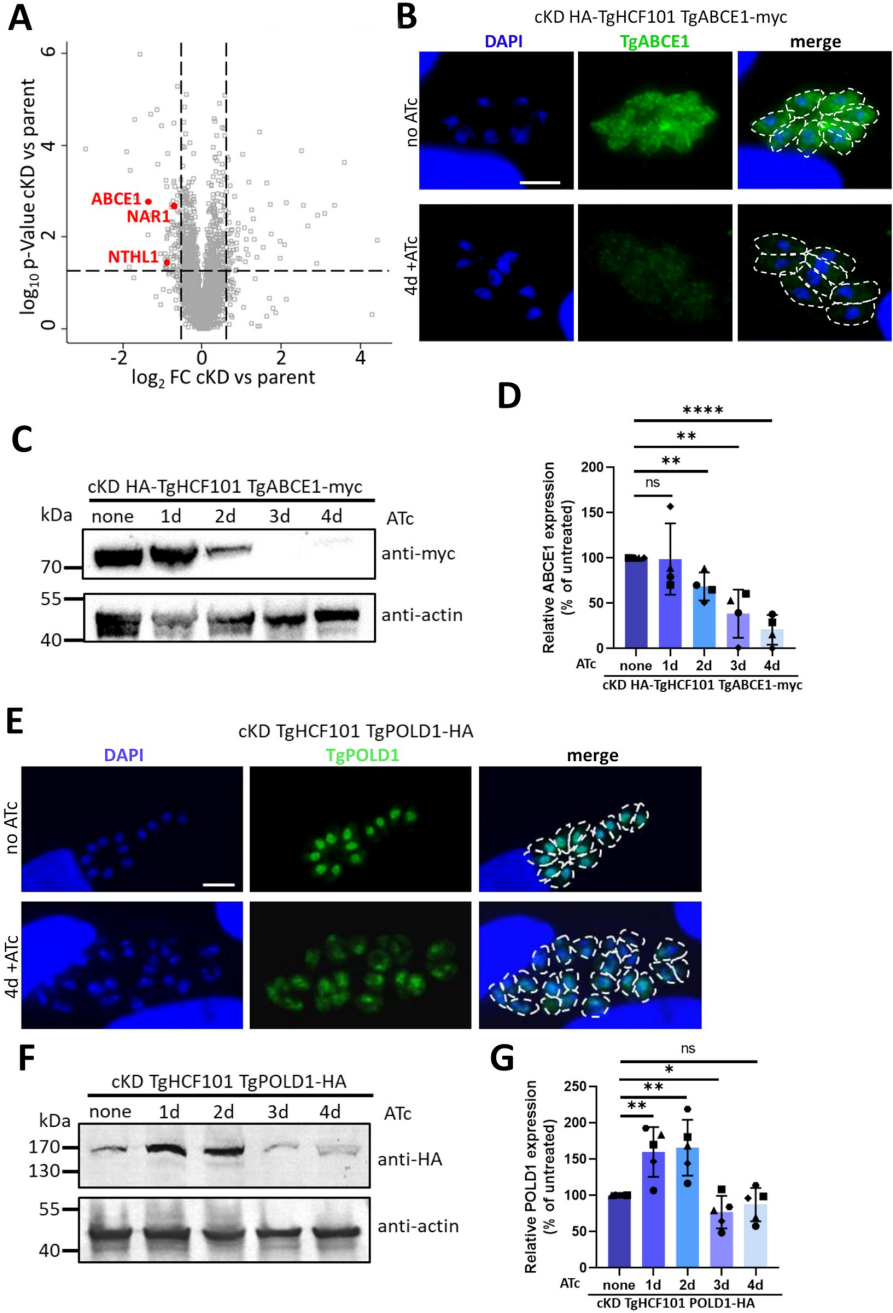
TgHCF101 is involved in Fe-S cluster biogenesis of the CIA pathway. **(A)** Volcano plot showing differential expression of proteins impacted by TgHCF101 depletion after 72 h of ATc treatment analyzed by label-free quantitative proteomic. Cut-offs were set at ≤1.5- or ≥1.5-fold change (FC) and *p*-value ≤0.05. Significant hits corresponding to predicted Fe-S cluster proteins are highlighted in red on the graph. **(B)** Immunofluorescence assay for the detection of myc-tagged TgABCE1 in the cKD TgHCF101 genetic background. Parasites were pre-incubated for 48 h in the presence of ATc and allowed to invade HFF-coated coverslips for another 48 h in the presence of ATc. The control (no ATc) was infected 24 h prior to fixation. TgABCE1 was detected with an anti-myc antibody and DNA was stained with DAPI. Parasites periphery is outlined by white dotted lines. Scale bar = 5 μm. **(C)** Immunoblot analysis of TgABCE1 abundance shows decrease upon TgHCF101 depletion following up to 4 days of treatment with ATc of the cKD HA-TgHCF101 TgABCE1-myc cell line. TgABCE1 was detected with anti-myc antibody and anti-actin antibody was used as a loading control. **(D)** Decrease of TgABCE1 expression upon TgHCF101 depletion was quantified by band densitometry analysis and normalized on the loading control of each respective lane. The relative abundance of TgABCE1 is presented as a percentage relative to the untreated control, set as 100%, for each biological replicate. Values are represented as the mean ± SD from *n* = 4 independent biological replicates, ** *p*-value ≤0.01, **** *p*-value ≤0.0001; ns, not significant (*p*-value ≥0.05), Student’s *t* test. **(E)** Immunofluorescence assay for the detection of HA-tagged TgPOLD1 in the cKD TgHCF101 genetic background. Parasites were pre-incubated for up to 4 days in presence of ATc on HFF-coated coverslips. The control (no ATc) was infected 24 h prior to fixation. TgPOLD1 was detected with an anti-HA antibody and DNA was stained with DAPI. Parasites periphery is outlined by white dotted lines. Scale bar = 5 μm. **(F)** Immunoblot analysis of TgPOLD1 abundance in conditions of TgHCF101 depletion following up to 4 days of treatment with ATc of the cKD TgHCF101 TgPOLD1-HA cell line. TgPOLD1 expression was detected with anti-HA antibody and anti-actin antibody was used as a loading control. **(G)** Changes in TgPOLD1 expression upon TgHCF101 depletion was quantified by band densitometry analysis and normalized on the loading control of each respective lane. The relative abundance of TgPOLD1 is presented as a percentage relative to the untreated control, set as 100%, for each biological replicate. Values are represented as the mean ± SD from *n* = 5 independent biological replicates, * *p*-value ≤0.05; ** *p*-value ≤0.01; ns, not significant (*p*-value ≥0.05), Student’s *t* test. The data underlying this figure can be found in S2 and S3 Tables and in S1 Data. cKD, conditional knock-down; CIA, cytosolic iron–sulfur cluster assembly; HA, hemagglutinin; HFF, human foreskin fibroblast.

Given the potential implication of TgHCF101 in the biogenesis of Fe-S proteins, we also specifically searched for putative Fe-S proteins in the less expressed proteins highlighted by the label-free quantitative proteomic analysis. We have previously estimated the Fe-S proteome using a computational predicting metal-binding sites in protein sequences [49], which, coupled with the data from global mapping of *T*. *gondii* proteins subcellular location by HyperLOPIT spatial proteomics [50], allows predicting client proteins present in the mitochondrion, apicoplast, or cytosol [19]. We found 3 Fe-S proteins that were less expressed upon TgHCF101 depletion (Fig 6A). Interestingly, they were all connected to the CIA pathway as they were NAR1, an Fe-S carrier to the CIA core machinery [15], and 2 Fe-S proteins depending on the CIA machinery for their maturation [51]: ABCE1 (Rli1 in yeast), a cytosolic ribosomal recycling factor and NTHL1, a nuclear DNA base-excision repair enzyme. In order to verify the proteomics data, we tagged TgABCE1 with a myc epitope in the context of the cKD HA-TgHCF101 mutant (S9A and S9B Fig). IFA showed a decrease in the cytosolic TgABCE1 signal (Fig 6B), which was supported by quantitative immunoblot analysis indicating a statistically significant decrease in TgABCE1 upon treatment of cKD HA-TgHCF101 mutant with ATc (Fig 6C and 6D).

We did not manage to tag TgNTHL1 to verify if its abundance was effectively affected by TgHCF101 depletion. However, the catalytic subunit of DNA polymerase delta (TgPOLD1), which is another nucleus-associated Fe-S protein, was found to be decreasing in abundance in the CIA-related TgABCB7L mutant [40]. Our quantitative proteomic analysis did not highlight a particular decrease in TgPOLD1 abundance upon TgHCF101 depletion: it was found to be stable, or even slightly more abundant (by a 1.4-fold), after 2 days of TgHCF101 depletion (S2 Table). Yet, we knew it could be tagged and decided to use this to investigate more thoroughly a potential global impact TgHCF101 depletion on nuclear and cytoplasmic Fe-S proteins. We thus created a conditional knockdown line of TgHCF101 in the POLD1-HA background [40] and verified that it displayed a similar phenotype to the original cKD HA-TgHCF101 cell line (S10 Fig). In the TgABCB7L mutant, TgPOLD1 abundance was found to be sharply decreasing early, before any noticeable impact on TgABCE1 [40]. In contrast, upon TgHCF101 depletion we observed a limited and delayed decrease in TgPOLD1 abundance (Fig 6E–6G) and intriguingly, TgPOLD1 levels even seemed to increase in the first 2 days of TgHCF101 depletion (Fig 6F and 6G). This is in sharp contrast with the more rapid and complete impact on TgABCE1 (Fig 6B–6D). Of note, IFAs highlighted that although TgHCF101 depletion did not lead to a marked loss of TgPOLD1, in some parasites the protein remained in the cytoplasm instead of being translocated to the nucleus (Fig 6E), which in fact corresponds to a stage where we have shown that DNA content of the parasite is affected (Fig 2G).

These results indicate that while perturbing TgHCF101 expression potentially has consequences on several proteins associated with the CIA machinery, it may be more specifically important for the maturation of the TgABCE1 cytosolic Fe-S protein.

### TgHCF101 is likely an Fe-S transfer protein of the CIA complex

**Fig 7:**
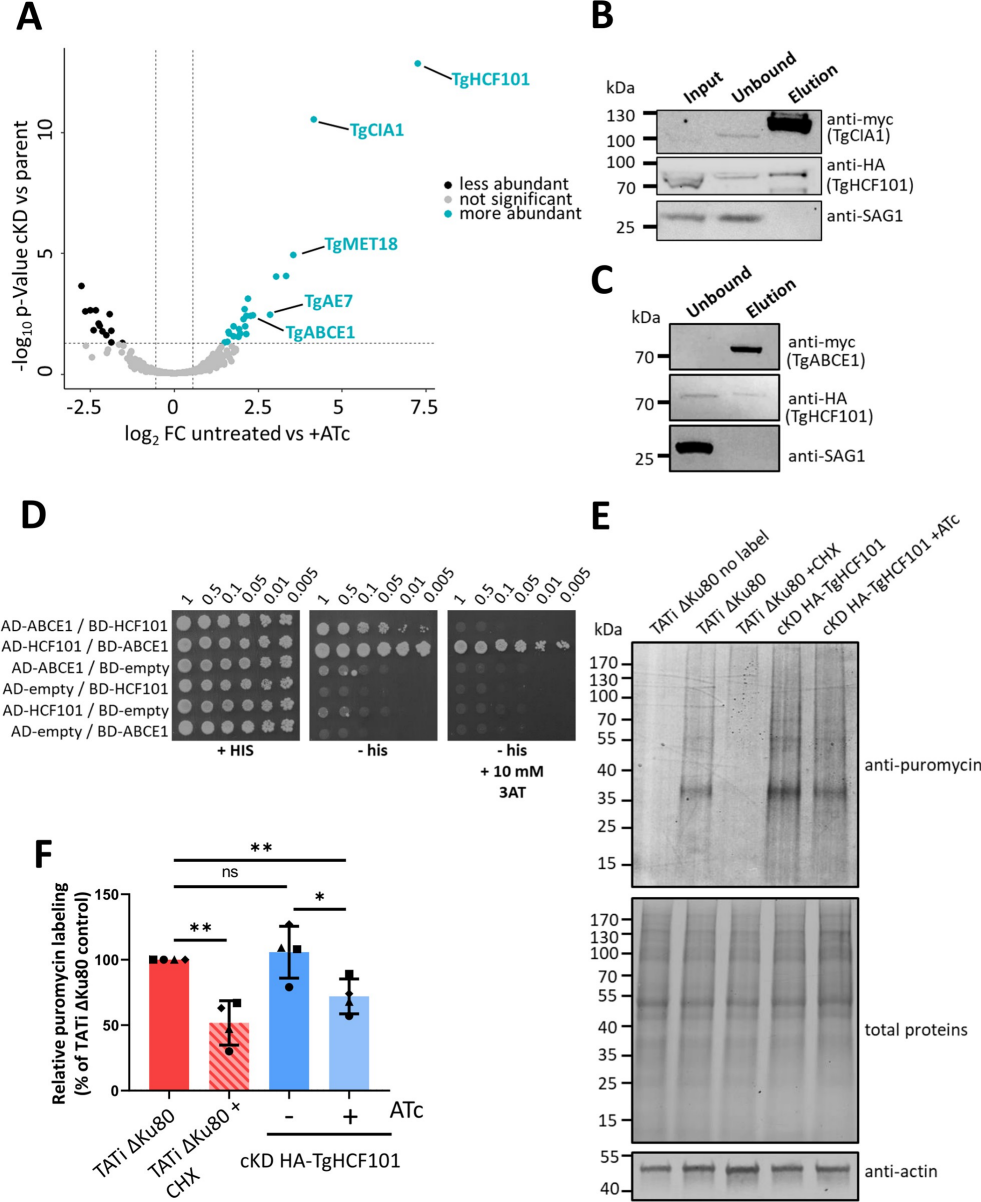
TgHCF101 is associated to the CIA targeting complex and specifically interacts with TgABCE1. **(A)** Volcano plot showing differential expression of TgHCF101 and co-immunoprecipitated proteins in the cKD HA-TgHCF101 cell line after TgHCF101 depletion (with 72 h of ATc treatment) or not, as analyzed by quantitative proteomic. Cut-offs were set at ≤1.5- or ≥1.5-fold change and *p*-value ≤0.05. Up-regulated proteins are highlighted in blue, significant hits corresponding to predicted proteins of the CIA targeting complex and target protein TgABCE1 were annotated on the graph. **(B)** Immunoblot analysis of a reverse co-immunoprecipitation assay of the myc-tagged TgCIA1 protein in the cKD HA-TgHCF101 background shows specific co-immunoprecipitation of TgHCF101, which is absent upon depletion by ATc. The anti-SAG1 antibody was used as a control for unspecifically bound proteins. **(C)** Immunoblot analysis of a reverse co-immunoprecipitation assay of the myc-tagged TgABCE1 protein showing TgHCF101 is co-immunoprecipitating. The anti-SAG1 antibody was used as a control for unspecifically bound proteins. **(D)** TgHCF101 interacts with TgABCE1 in a Gal4-based yeast two-hybrid assay. YRG2 cells co-transformed with AD- and BD-fusion proteins were grown to stationary phase, then serially diluted to OD_600_ values ranging from 1 to 5 × 10^−3^ before being spotted onto a control plate (+HIS) to assess cell viability, and a yeast two-hybrid test plate (-HIS) to assess interaction. Plates were incubated at 30°C, and yeast growth was recorded after 5 days. TgHCF101 exhibited a strong interaction with TgABCE1, independent of cloning orientation. The strongest interaction was observed between AD-TgHCF101 and BD-TgABCE1, with co-transformed cells growing at the lowest dilution tested in the presence of 10 mM 3-aminotriazole (3AT) as a competitive inhibitor. Neither TgHCF101 nor TgABCE1 alone showed HIS3 transactivation in the presence of 3AT. These results are representative of 3 independent experiments. **(E)** Immunoblot analysis of puromycin incorporation in the parental (TATi ΔKu80) and cKD TgHCF101 cell lines untreated or treated with ATc for 72 h. TATi ΔKu80 treated with cycloheximide (CHX) was used as a control for translation inhibition. The puromycin signal was detected with anti-puromycin antibody, and total protein content was visualized by stain-Free imaging technology. Anti-actin antibody was also used as a loading control. **(F)** Variation of puromycin incorporation in the different conditions was quantified by band densitometry and normalized on the total protein content of each respective lane. Puromycin labeling is presented as a percentage relative to the untreated control, set as 100% for each biological replicate. Values are represented as the mean and SD of 4 independent biological replicates; * *p*-value ≤0.05; ** *p*-value ≤0.01; ns, not significant (*p*-value ≥0.05), Student’s *t* test. The data underlying this figure can be found in S4 Table and in S1 Data. cKD, conditional knock-down; CIA, cytosolic iron–sulfur cluster assembly; HA, hemagglutinin.

To get further insights on the role played by TgHCF101 in the CIA machinery, we next performed co-immunoprecipitations (co-IPs) and mass spectrometry identification of associated proteins, comparing lysates of cKD HA-TgHCF101 parasites grown in the presence of ATc or not (S4 Table). Strikingly, we pulled-down all 3 *T*. *gondii* homologs of the core components of the CTC: MET18, CIA1, and AE7 (an MIP18 family protein) (Fig 7A). We managed to tag the TgCIA1 protein with a myc epitope tag in the context of the HA-tagged TgHCF101 cell line (S9C and S9D Fig) and performed a reverse IP by which we managed to specifically pull-down TgHCF101, confirming their interaction (Fig 7B). It should be noted that this result was only obtained when this experiment was performed after chemical crosslinking, suggesting a possibly indirect or transient interaction between the proteins.

Another interesting candidate that was found co-immunoprecipitated with TgHCF101 was TgABCE1, strengthening the possibility of an important relationship between the 2 proteins. We next performed a reverse IP and could recover small amounts of TgHCF101 co-eluting specifically with TgABCE1 (Fig 7C). To further investigate this potential interaction, we performed yeast two-hybrid assay, based on interaction-dependent transactivation of the *HIS3* reporter gene (Fig 7D). This experiment confirmed that there is a direct interaction between TgHCF101 and TgABCE1, and highlighted a particularly strong interaction, as it was retained when using 3-aminotriazole, a competitive inhibitor of the *HIS3* gene product (Fig 7D). While this confirms that TgABCE1 is likely a client protein of TgHCF101, we could not confirm a direct interaction between TgHCF101 and TgNTHL1 using the same method (S11 Fig). Given the known implication of ABCE1 in translation, we next evaluated the impact of TgHCF101 depletion on translation by using a puromycin-based assay (Fig 7E). Puromycin can mimic aminoacyl-tRNA and becomes covalently attached to nascent peptides, thereby allowing the evaluation of protein elongation rates with an anti-puromycin antibody [52]. Using this method, we could show that TgHCF101 depletion leads to a modest but a statistically significant decrease in the overall translation rate (Fig 7E and 7F).

Together, these results suggest that TgHCF101 acts as an Fe-S transfer protein to the translation regulator TgABCE1.

## Discussion

Fe-S clusters are universal among living organisms, where they play key roles in many important biological processes as cofactors of proteins involved for instance in housekeeping functions like respiration, photosynthesis, as well as genome expression or maintenance [6]. These cofactors were acquired early during evolution, and thus they played a fundamental role in the evolution of the eukaryotic cells as they were inherited through endosymbiosis to organelles such as the mitochondrion or plastids [5]. Different Fe-S cluster synthesis pathways show globally conserved mechanistic and biochemical features to assemble and transfer the clusters prior to transferring them to client proteins [7]. However, beyond a core Fe-S synthesizing machinery, given the large diversity of eukaryotic lineages, it is likely that some have evolved some specific features.

**Fig 8:**
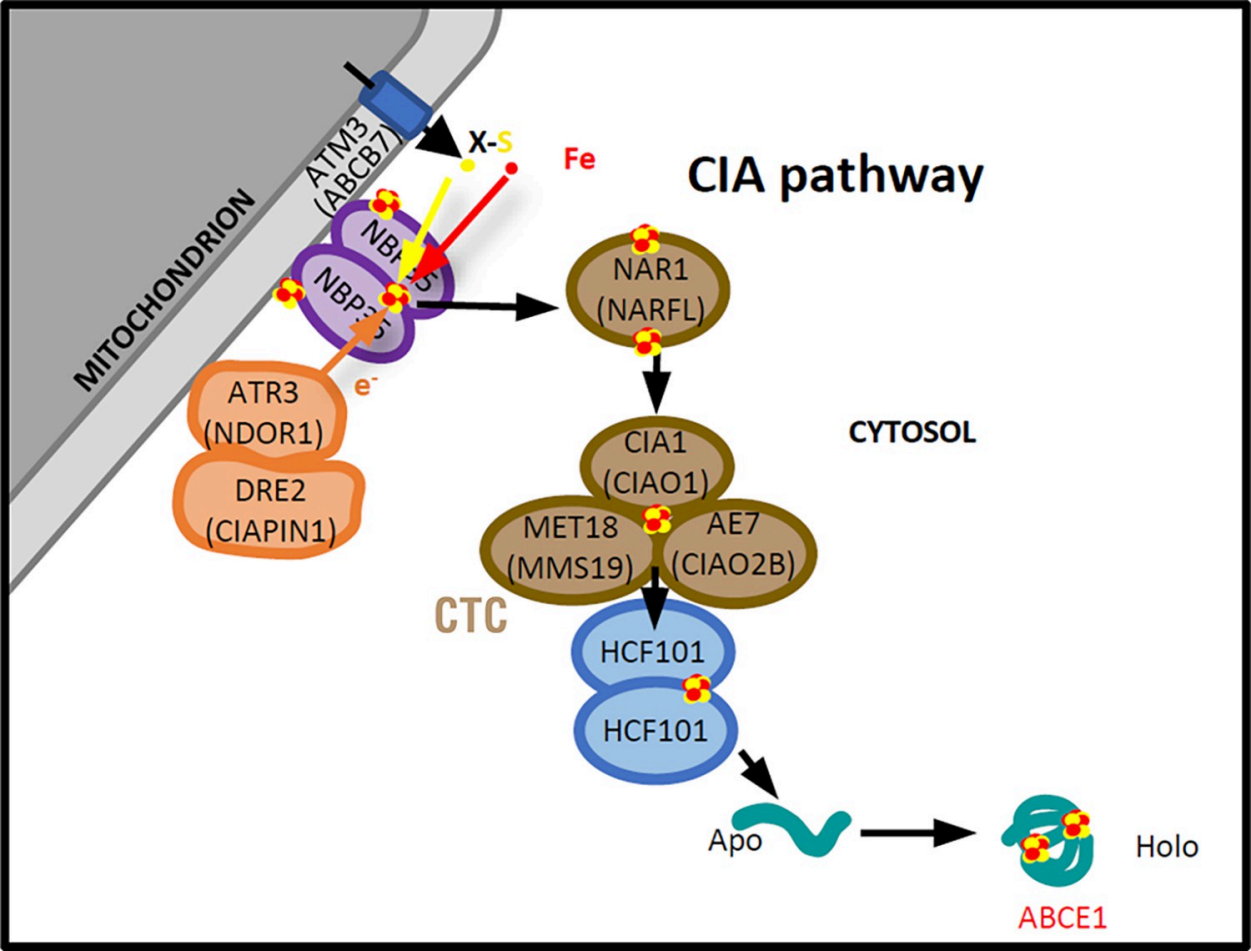
Schematic representation of the putative organization of the CIA pathway in *T*. *gondii*. This scheme places TgHCF101 as an Fe-S transfer protein from the CTC to client protein ABCE1. Plant nomenclature was used for the purpose of the figure, but names for human homologs are mentioned between brackets when appropriate. CIA, cytosolic iron–sulfur cluster assembly; CTC, CIA targeting complex.

For instance, proteins of the MRP/NBP35 family (NBP35, Cfd1, Ind1, and HCF101) have in common a central P-loop NTPase domain [27], but are either involved in scaffolding or transfer of Fe-S clusters at specific subcellular locations like the mitochondrion, the plastid, or in the cytosol [12,25,26,53]. Presence of MRP family members in bacteria that are able to bind Fe-S clusters point to ancient and conserved function linked to Fe-S biogenesis for these proteins [54]. Another feature shared by members of this family is their ability to form homo- or hetero-dimers bridging one Fe-S cluster at their interface through 2 conserved and functionally essential Cys residues [55]. Interestingly, HCF101 also presents at its N-terminal an MIP18-like domain that constitutes most of a bacterial protein called SufT that is involved in Fe-S maturation [29,54,56], as well as a C-terminal domain whose function is not yet defined (DUF971). The presence of additional domain besides the conserved NTPase core domain in members of the MRP family like HCF101 suggests that they may have additional or more specialized functions. Another clue of evolutionary specialization comes from the different localization of protein isoforms in different species, hinting for a possible association to different Fe-S cluster biogenesis machineries. A recent study suggested a wide diversity in localization for HCF101 paralogs across eukaryotes, with isoforms potentially localizing to the mitochondrion in *Tetrahymena* or in the cytosol in *T*. *gondii* [24]. More precisely, in some chromalveolates a mitochondrial HCF101 isoform likely replaced Ind1 for the maturation of Fe-S subunits of the complex I of the mitochondrial electron transport chain. Yet, as *T*. *gondii* lacks complex I, it has no Ind1 homolog and no particular need for a mitochondrion-located HCF101 isoform either. Moreover, while HCF101 was initially characterized as a chloroplast-associated protein with a specialized function in maintaining PSI homeostasis, our study confirms that although *T*. *gondii* harbors a plastid, TgHCF101 is expressed in the cytosol (Figs 1F and S2A), which is consistent with the absence of photosynthesis-related pathways in *T*. *gondii*. Our study establishes for the first time that the cytosolic expression of TgHCF101 is functionally related to the CIA pathway (Fig 8).

Previous functional investigations of the CIA pathway in *T*. *gondii* focused on TgABCB7L, a mitochondrial transporter presumably involved in providing a sulfur-containing precursor for further processing by the cytosolic Fe-S assembly machinery [40] and on TgNBP35, an Fe-S scaffolding protein of the CIA that displays an unusual association with the outer mitochondrial membrane in the parasites [20]. The mitochondrial localization of these proteins, which are quite upstream in the CIA machinery, suggests that the assembly of cytosolic Fe-S clusters likely happens at the cytosolic face of the mitochondrion in *T*. *gondii*, and then probably shuttle through the *T*. *gondii* NAR1 homolog to the CTC for subsequent transfer to client proteins (Fig 8). CTC proteins have so far not been functionally investigated in the parasite, but both TgABCB7L and TgNBP35 are essential for parasite growth and were both shown to be important for TgABCE1 stability [20,40]. The TgNAR1 mutant that we generated in this study also shows that this CIA shuttle protein is essential for parasite fitness (S7D Fig). Combining genome-wide data for potential essentiality and localization [50,57] to metal-binding site prediction algorithms [49], our previous assessment of the putative *T*. *gondii* cytosolic and nuclear Fe-S proteome highlighted proteins involved in key functions like genome reparation and maintenance, mRNA synthesis, and protein expression [19]. It is thus unsurprising to see that *T*. *gondii* mutants of the CIA pathway are severely impaired in growth. Our quantitative proteomics analysis of the TgHCF101 mutant did not reveal an impact on a large number of nuclear or cytosolic Fe-S proteins, which may indicate that this protein could be involved in cluster transfer to a reduced subset of Fe-S proteins. Besides the decrease in abundance of TgNAR1, strengthening the evidence of TgHCF101 involvement in the CIA pathway, quantitative proteomics data revealed a decrease in 2 potential client Fe-S proteins: TgABCE1 and TgNTHL1. While we could establish a direct association of TgHCF101 with TgABCE1 by co-immunoprecipitations and yeast two-hybrid (Fig 7A, 7C, and 7D), we did not manage to tag TgNTHL1 to perform co-immunoprecipitations and we did not obtain proof of a direct TgHCF101/TgNTHL1 interaction by yeast two-hybrid. Besides, when we tested the impact of TgHCF101 depletion on TgPOLD1, an Fe-S protein known to be affected by the disruption of the CIA pathway [40], we saw a limited and delayed effect compared with the one we observed on TgABCE1 (Fig 6). Whether or not TgHCF101 is able to transfer clusters to Fe-S proteins besides TgABCE1 is still an open question, yet, so far, the evidence we gathered points primarily to TgABCE1 as its main client protein.

Our phenotypic analysis of TgHCF101-depleted parasites revealed cell division and DNA replication problems (Fig 2D and 2G) that might result from an impact on Fe-S protein-mediated DNA maintenance, but may also be secondary (and more general) effects of TgHCF101 depletion. In addition, through perturbation of TgABCE1 function, we measured a decrease in the overall translation rate in the parasites (Fig 7E and 7F) that could contribute to their decreased fitness and growth problems. Upon 2 days of TgHCF101 depletion, parasites also started to display an accumulation of LDs (Fig 3). LDs are known to be induced as part of an integrated stress response to cellular injuries and may be induced in conditions such as oxidative stress [43]. For instance, during the particular Fe-induced cell death called ferroptosis, iron excess combined to oxygen leads to the accumulation of reactive oxygen species (ROS). This leads to subsequent peroxidation of lipids, which in turn induces the accumulation of lipid droplets that act as antioxidant organelles to control polyunsaturated fatty acid storage in triglycerides in order to reduce membrane lipid peroxidation [58]. Although it is possible that perturbation of the CIA pathway created local imbalance in cytosolic Fe concentration and ROS-dependent stress that contributed to the demise of the parasites, it is to note that our previous work has shown that general Fe deprivation has a similar LD-inducing effect on the parasites [59]. So, altogether, this may rather suggest that specific CIA-dependent Fe-S protein(s) that remain to be identified could be involved in regulating LDs. Even more so than the induction of LDs, initiation of stage conversion to the bradyzoite persistence form is a hallmark of the stress response in *T*. *gondii* [37]. Upon depletion of TgHCF101, we could detect early signs of differentiation (Fig 5), but the parasites were largely unable to progress to full stage conversion and died instead (Figs 2C and 5E). Importantly, overall our data show that TgHCF101 is essential for parasite viability, and although our findings point towards an involvement of this protein in the eukaryote-conserved CIA pathway, its specific absence from the mammalian hosts of the parasite makes it a good potential drug target. This calls for further structure/function studies of TgHCF101 in order to potentially design specific inhibitors.

There is a vast repertoire of Fe-S client proteins with a diverse range of structures and roles, and one important conundrum remaining in the Fe-S research field is resolving functional specificity of the transfer proteins during the addition of Fe-S to apoproteins. Key investigations in budding yeast allowed the identification of mutually exclusive sub-complexes of the CTC that can transfer Fe-S clusters to different proteins [51,60]. In particular, it was shown that a complex of 2 small proteins, Yae1 and Lto1, functions as a target-specific adaptor to recruit the yeast homolog of ABCE1 (called Rli1) to the generic CIA machinery [60]. These adaptor proteins are well-conserved in phylogenetically close opisthokonts like humans, in which they have also been shown to be important for ABCE1 maturation [61,62]. However, Yae1 and Lto1 are clearly not conserved across all eukaryotes but found essentially in fungi, metazoan, and plants (S12 Fig), and thus noticeably they do not seem to have homologs in the Alveolata superphylum that includes apicomplexan parasites. So, while ABCE1 is arguably one of the most conserved proteins in evolution and is universally present in all eukaryotes [63], the factors driving its CIA-dependent maturation seem phylogenetically divergent. We have now established that in *T*. *gondii*, HCF101 is associated to the CTC and likely plays that role in this organism. Given sequence conservation of HCF101 homologs, it is also likely to be the case in other apicomplexan parasites and even possibly in one isoform present in the photosynthetic relative *C*. *velia* (Figs 1A, 1B, and S1). Our results highlight the complex evolutionary adaptation that accompanied the maturation of eukaryotic Fe-S proteins and calls for in-depth structural analysis of this interaction. Not only this would provide invaluable evolutionary insights into the molecular machinery supporting Fe-S cluster transfer, but also may provide new prospects for interfering specifically with an essential function as a new strategy against Apicomplexa-caused diseases.

## Materials and methods

### Cell culture

*Toxoplasma gondii* RH tachyzoites and derived transgenic cell lines generated in this study were routinely maintained through passages in human foreskin fibroblasts (HFFs) monolayer (ATCC CRL-1634). HFFs and parasites were cultured in standard Dulbecco’s Modified Eagle Medium (DMEM, supplemented with 5% decomplemented fetal bovine serum (FBS), 2 mM L-glutamine, 100 U/ml penicillin, and 100 μg/ml streptomycin (Gibco) in a controlled atmosphere at 37°C and with 5% CO_2_.

For immunoprecipitation assays, parasites were grown in Vero cells (ATCC, CCL-81) in the same conditions as for the HFFs.

Transgenic cell lines were generated in the TATi ΔKu80 genetic background line lacking the *Ku80* gene and expressing the TATi transactivator required for the TetOff system [64]. For positive selection of transgenic parasites bearing resistance cassettes for expressing dihydrofolate reductase thymidylate synthase (DHFR-TS) or chloramphenicol acetyltransferase (CAT) were grown with 1 μm pyrimethamine or 20 μm chloramphenicol (Sigma-Aldrich, SML3579, C0378), respectively. Conditional depletion in the TetOff conditional knockdown lines was achieved by incubation with 0,5 μg/ml anhydrotetracycline (ATc, Fluka 37919) for the indicated duration of the assay.

### Bioinformatic analyses

Genomic and protein sequences were retrieved from http://www.toxodb.org or https://www.ncbi.nlm.nih.gov/protein/. Protein alignments were performed from eukaryotic MRP family proteins (S1 Table), whose sequences were obtained from Grosche and colleagues [65], and updated by manual curation. Iterative rounds of alignment were performed with MAFFT v.7.475 using the E-INF-i refinement method (adapted for several conserved motifs that are embedded in long unalignable regions) https://mafft.cbrc.jp/alignment/server/index.html [66]. Sequence trimming and optimization was then performed with BMGE v.1.12 (https://ngphylogeny.fr/tools/tool/273/form) [67], with a minimal block size of 1. A phylogeny was then inferred from this data set by maximum likelihood using the IQ-TREE software v.2.1.2 (http://www.iqtree.org/) [68], with 1,000 ultrafast bootstrap replicates [69]. Phylogenetic trees were rendered using the MEGA software v.11.0.13 (https://www.megasoftware.net/) [70] or with iTOL (https://itol.embl.de/) [71]. Tree files are available at https://doi.org/10.6084/m9.figshare.28238195.

Transit peptide and subcellular localization predictions were performed using the ChloroP 1.1 (https://services.healthtech.dtu.dk/services/ChloroP-1.1/), IPSORT (http://ipsort.hgc.jp/), and Localizer 1.0.4 (http://localizer.csiro.au/) algorithms. Domain searches were performed using the Interpro database (https://www.ebi.ac.uk/interpro/).

### Generating a GFP-tagged TgHCF101 cell line

cDNA corresponding to the *TgHCF101* gene (TGGT1_318590) was amplified by PCR with primers ML4715 and ML4716 (all primers used in the present study are listed in S5 Table) and sub-cloned using XhoI and KpnI into the pEZS-NL vector (D. Ehrhardt, https://deepgreen.dpb.carnegiescience.edu/cell%20imaging%20site%20/html/vectors.html) for a C-terminal GFP fusion. The TgHCF101-GFP cassette was then amplified by PCR using the ML4817 and ML4818 primers and cloned using BclI and EcoRV in a BglII/EcorV-digested pTUB-IMC1-TdT vector [72] to drive the expression from a tubulin promoter. Tachyzoites were transfected with 100 μg of plasmid and observed by fluorescence microsocopy.

### Generating a conditional TgHCF101 knock-down cell line

To generate the construct for the tetracycline-regulated conditional depletion of TgHCF101 and add an N-terminal HA tag, we digested the DHFR-TetO7Sag4 plasmid [72] by BglII and inserted a fragment coding for a single HA tag generated through hybridization of the ML4924 and ML4925 oligonucleotides. Then, a 1,066 bp fragment corresponding to the 5′ coding part of TgHCF101 was amplified by PCR with primers ML4926 and ML4927 and inserted after digesting with BglII and NotI, to yield the DHFR-TetO7Sag4-HA-TgHCF101 plasmid. The TATi ΔKu80 cell line was transfected with 80 μg of the BsiWI-linearized plasmid. Transgenic parasites, named cKD HA-TgHCF101, were selected with pyrimethamine and cloned by limiting dilution. Positive clones were verified by PCR with primers ML1771 and ML6043.

### Generating complemented cell lines

The cKD TgHCF101-HA cell line was complemented by adding an extra copy of the *TgHCF101* gene under the control of a tubulin promoter at the *UPRT* locus. The entire *TgHCF101* cDNA sequence (1,935 bp) was amplified by PCR using primers ML6409/ML6410 from the plasmid pGBKT7-myc-HCF101 to express an N-terminal myc-tagged HCF101 copy. This sequence was then cloned downstream of the tubulin promoter in the pUPRT-TUB vector [64], resulting in the pUPRT-myc-TgHCF101 plasmid. The plasmid was subsequently linearized prior to transfecting the mutant cell line, together with a plasmid expressing Cas9 and a *UPRT*-specific guide RNA (gRNA) under the control of a U6 promoter [73]. Transgenic parasites were selected using 5 μm 5-fluorodeoxyuridine (Sigma-Aldrich) and cloned by serial limiting dilution to obtain the cKD TgHCF101 WT complemented cell line.

For complementation with the copy of TgHCF101 lacking the LEW tripeptide motif, the cDNA sequence was amplified by PCR using primers ML6409/ML6412 from the plasmid pGADT7-myc-HCF101, to remove the tripeptide motif in the C-terminal region. All constructs were verified by sequencing. Cloning and transfection were performed as previously described to establish the cKD TgHCF101 -LEW comp cell line. For both cell lines, correct integration at the *UPRT* locus was verified by PCR using primers ML6044 and ML6396.

### Generating a conditional TgNAR1 knock-down cell line

We digested the DHFR-TetO7Sag4 plasmid by BglII and then, a fragment corresponding to the 5′ coding part of *TgNAR1* was amplified by PCR with primers ML5862 and ML5863 and inserted after digesting with BamHI and NotI to yield the DHFR-TetO7Sag4-TgNAR1 plasmid. The TATi ΔKu80 cell line was transfected with 80 μg of the NsiI-linearized plasmid. Transgenic parasites, named cKD HA-TgNAR1, were selected with pyrimethamine and cloned by limiting dilution. Positive clones were verified by PCR with primers ML1771 and ML5947.

### Tagging of ABCE1 and CIA1 in the TgHCF101 knock-down background

A CRISPR-based strategy was used to C-terminally tag proteins of interest in the cKD HA-TgHCF101 background. gRNAs targeting the C-terminal end of the gene of interest were selected using CHOP-CHOP tool (https://chopchop.cbu.uib.no/). gRNAs were cloned into the pU6-Cas9 Universal Plasmid (Addgene, 52694) using BsaI. Donor DNA was amplified by PCR using the high fidelity KOD DNA polymerase (Novagen) to amplify a fragment containing the tag and the resistance cassette, using the pLIC-myc-CAT plasmid as a template, adding at both ends 30-nucleotide overhangs homologous to the C-terminal region of the gene of interest for homologous recombination. *TgABCE1* (TGGT1_216790) tagging was performed by using primers ML5126 and ML5127 for the gRNA and ML5128 and ML5129 to amplify donor DNA, yielding the cKD HA-TgHCF101 TgABCE1-myc cell line. Integration of the construct was controlled by PCR using GoTaq DNA polymerase (Promega) with primers ML6115 and ML4310. *TgCIA1* (TGGT1_313280) tagging was performed by using primers ML5932 and ML5933 for the gRNA and ML5934 and ML5935 to amplify donor DNA, yielding the cKD HA-TgHCF101 TgCIA1-myc cell line. Integration of the construct was controlled by PCR using GoTaq DNA polymerase (Promega) with primers ML5936 and ML4310.

### Generating a TgHCF101 knock-down cell line with HA-tagged POLD1

The POLD1-HA cell line [40] was used as a background to generate a tetracycline-regulated conditional TgHCF101 mutant. Primers ML4926 and ML4927 were used to amplify a 1,066 bp 5′ fragment of the *TgHCF101* coding sequence and cloned using BglII/NotI in the DHFR-TetO7Sag4 plasmid [72] to yield the DHFR-TetO7Sag4-TgHCF101 plasmid. The POLD1-HA cell line was transfected with 80 μg of this plasmid linearized by NsiI. Transgenic parasites were selected with pyrimethamine and cloned by limiting dilution. Positive clones were verified by PCR with primers ML2456 and ML6043.

### Semi-quantitative RT-PCR

Semi-quantitative RT-PCR was performed as described previously [74]. Briefly, 1 μg of RNA was used as a template for each RT-PCR reaction and specific primers for *TgHCF101* (ML5112/ML5113), *TgNAR1* (ML6467/ML6468), or *TUB2* (Tubulin β chain) (ML841/ML842) were used. Twenty-four cycles of PCR were performed.

### Immunofluorescence assays

For IFAs, coverslips seeded with HFFs were infected by *T*. *gondii* tachyzoites. Intracellular parasites and host cell monolayer were then fixed with 4% (w/v) paraformaldehyde (PFA, diluted in phosphate-buffered saline, PBS) for 20 min. After fixation, cells were permeabilized with 0.3% (v/v) Triton X-100 (diluted in PBS) for 10 min. Coverslips were blocked with 2% (w/v) bovine serum albumin (BSA) for 1 h prior to immunolabeling with primary antibody for 1 h. After 3 washes in PBS, corresponding secondary antibody was incubated for 1 h. Coverslips were finally incubated with 1 μg/ml 4,6-diamidino-2-phenylindole (DAPI) for 5 min before 3 washes in PBS and, lastly, mounted using Immu-Mount (Thermo Fisher) onto microscope slides. Primary antibodies used were prepared in 2% BSA (diluted in PBS) and used at the following concentrations: rat monoclonal anti-HA (1:1,000, 3F10 Roche), mouse monoclonal anti-myc (1:100, 9E10 Sigma), rabbit anti-IMC3 (1:1,000) [75], mouse anti-SAG1 (T41E5, 1:1,000) [76], rabbit anti-armadillo repeats only (ARO, 1:1,000) [48], rabbit anti-PDH-E2 (1:500) [22], mouse anti-F1β ATPAse (1:1,000, gift of P. Bradley). Highly cross-adsorbed Alexa Fluor 488-conjugated or Alexa Fluor 594-conjugated anti rat-, rabbit-, or mouse-IgG secondary antibodies were all from Thermo Fisher and were diluted at 1:4,000. Cysts were stained with biotin labeled *Dolichos Biflorus* lectin (1:300, Sigma L-6533) and detected with FITC-conjugated streptavidin (1,300, Invitrogen, SNN1008). Lipid droplets were detected with Nile Red (1 μg/ml, Sigma 72485), using the CY3/DsRed BP550/25 FT570 BP605/70 filter cube (Zeiss) on an epifluorescence microscope.

All images were acquired at the Montpellier Ressources Imagerie (MRI) facility. Observations were performed with Zeiss AxioImager Z1 and Z2 epifluorescence microscopes equipped with a Zeiss Axiocam MRm CCD camera and 63×/1.4 or 100×/1.4 Oil Plan Achromat objective. Images were processed on Zen Blue v3.6 (Blue edition) software (Zeiss). Z-stack acquisitions were processed by maximum intensity orthogonal projection when assessing lipid droplets number and area. Adjustments of brightness and contrast were applied uniformly and paired images were acquired with the same exposure time.

### Plaque and replication assays

The lytic cycle of tachyzoites was assessed by plaque assay as described previously [59]. Briefly, tachyzoites from the cKD HA-TgHCF101 transgenic cell line or parental strain (TATi ΔKu80) were allowed to invade monolayers of HFFs in the presence or absence of ATc. Parasites were cultivated for 7 days at 37°C and 5% CO_2_ and fixed with 4% (w/v) PFA (diluted in PBS) for 20 min. Cells were stained with 0.1% crystal violet solution (V5265, Sigma-Aldrich), washed, and air-dried before imaging on an Olympus MVX10 microscope. For the reversibility assay, drug washout was carefully performed after 7 days of ATc pretreatment and cultures were kept for another 7 days of growth, non-treated control conditions were also infected at this time point.

For replication assay, parasites were pretreated in flasks for 48 h with ATc and extracellular parasites were allowed to invade HFF monolayer on coverslips for another 24 h before being fixed and performing parasites immunodetection by IFA with mouse anti-SAG1 antibody as described before [72]. The number of parasites per vacuole was scored. Independent experiments were conducted 3 times, and 200 random vacuoles were counted for each condition.

### Immunoblotting and antibodies

Protein extracts were prepared from 10^7^ extracellular parasites resuspended in Laemmli buffer at a final concentration of 10^6^ parasites/μl. Extracts were treated with benzonase to remove DNA from samples and resolved by SDS-PAGE before being transferred on nitrocellulose membrane for subsequent protein detection. Primary antibodies used for immunodetection were resuspended at their respective working concentration in 5% (w/v) milk in TNT buffer (0.1 M Tris-HCl (pH 7.6), 0.15 M NaCl, 0.05%, Tween 20). Antibodies used in this study for immunoblot detection were rat anti-HA (1:1,000, Roche), mouse anti-myc (1:100, Sigma), mouse anti-SAG1 (1:50, hybridoma), mouse anti-actin (1:25, hybridoma) [77], mouse anti-ROP7 (1:1,000, T43H1) [78], mouse anti-puromycin (1:1,000, 12D10, MABE943 Sigma), and rabbit anti-lipoic acid (1:500, ab58724 Abcam) [79]. Alkaline phosphatase-conjugated anti mouse- or anti rabbit-IgG were from Promega and used at 1:2,500. Alkaline phosphatase-conjugated anti rat-IgG secondary antibodies were from Sigma and used at 1:2,500. Horseradish peroxidase-conjugated anti rat-, rabbit-, or mouse-IgG secondary antibodies were all from Thermo Fisher and were diluted at 1:10,000.

### Puromycin labeling

Strains of interest were grown for 3 days in the presence or absence of ATc. Freshly egressed parasites were filtered on 40 μm Cell Strainer (VWR, 723–2757). After filtration and counting, parasites were treated with puromycin (100 μg/ml, puromycin dihydrochloride, Sigma) for 15 min at 37°C and 5% CO_2_. For translation inhibition control, parasites were treated with cycloheximide (100 μg/ml, Sigma) for 10 min prior to puromycin incubation. After treatment, parasites were washed in DPBS (Dulbecco’s phosphate-buffered saline, Gibco) and collected by centrifugation. Pellet was resuspended in Laemmli buffer and separated on Mini-Protean TGX Stain-free gels 12% (BioRad) activated by a UV-induced 1-min reaction to produce tryptophan residue fluorescence in order to allow for global protein quantification, following manufacturer instructions. Proteins were then transferred to nitrocellulose membrane for immunodetection using mouse anti-puromycin (1:1,000, 12D10, MABE943 Sigma) and mouse anti-actin (1:25, hybridoma). Total protein content was assessed by Stain-free detection and puromycin signal was detected with secondary antibody mouse coupled with alkaline phosphatase. Both signals were quantified by densitometry using the ImageJ software.

### Electron microscopy

Parasites were pretreated with ATc for 48 h and allowed to reinvade for 24 h in the presence of ATc. Untreated parasites were used as a control for normal morphology. Cells were then fixed with 2.5% glutaraldehyde in cacodylate buffer 0.1 M (pH 7.4). Coverslips were subsequently processed using a Pelco Biowave pro+ (Ted Pella). Samples were postfixed in 1% OsO4 and 2% uranyl acetate, dehydrated in acetonitrile series and embedded in Epon 118 using the following parameters: Glutaraldehyde (150 W ON/OFF/ON 1-min cycles); 2 buffer washes (40 s 150 W); OsO4 (150 W ON/OFF/ON/OFF/ON 1-min cycles); 2 water washes (40 s 150 W); uranyl acetate (100 W ON/OFF/ON 1-min cycles); dehydration (40 s 150 W); resin infiltration (350 W 3-min cycles). Fixation and infiltration steps were performed under vacuum. Polymerization was performed at 60°C for 48 h. Ultrathin sections at 70 nM were cut with a Leica UC7 ultramicrotome, counterstained with uranyl acetate and lead citrate and observed in a Jeol 1400+ transmission electron microscope from the MEA Montpellier Electron Microscopy Platform. All chemicals were from Electron Microscopy Sciences and solvents were from Sigma.

### Label-free quantitative proteomics

Parasites from the TATi ΔKu80 and cKD HA-TgHCF101 cell lines were treated for 72 h in HFF seeded in T75 cm^2^ flasks. After suitable incubation, parasites were released from host cells with a cell scraper and passed through a 25G needle before filtration on a fiber glass wool column. Parasites were pelleted and washed in Hank’s balanced salt solution (HBSS, Gibco). Parasites were resuspended in lysis buffer (1% SDS, 50 mM Tris HCL (pH 8), 10 mM EDTA (pH 8)) and protein quantification was determined with the bicinchoninic acid kit (Abcam). For each condition, 20 μg of protein resuspended in Laemmli buffer were resolved on a 10% SDS-PAGE for 35 min at 100V. Proteins were fixed with a combination of acetic acid and ethanol and stained in PageBlue Protein Staining Solution (Thermo Scientific). Each lane was cut in 3 identical pieces which were digested with trypsin and peptide extraction was done as previously described [80].

LC-MS/MS experiments were performed using an Ultimate 3000 RSLC nano system (Thermo Fisher) interfaced online with a nano easy ion source and an Exploris 240 Plus Orbitrap mass spectrometer (Thermo Fisher). The.raw files were analyzed with MaxQuant version 2.0.3.0 using default settings (PMID: 19029910). The minimal peptide length was set to 6. The files were searched against the *T*. *gondii* proteome (March 2020, https://www.uniprot.org/proteomes/UP000005641-8450). Identified proteins were filtered according to the following criteria: at least 2 different trypsin peptides with at least 1 unique peptide, and a protein E value smaller than 0.01 were required. Using the above criteria, the rate of false peptide sequence assignment and false protein identification were lower than 1%. Proteins were quantified by label-free method with MaxQuant software using unique and razor peptides intensities [81]. Statistical analyses were carried out using RStudio package software. The protein intensity ratio (protein intensity in mutant/protein intensity in parent) and statistical tests were applied to identify the significant differences in the protein abundance. Hits were retained if they were quantified in at least 3 of the 4 replicates in at least 1 experiment. Proteins with a statistically significant (*p* < 0.05 or 0.01 with or without Benjamini correction) quantitative ratio were considered as significantly up-regulated and down-regulated, respectively. Volcano plots were generated with the Perseus software v2.0.7.0 (https://maxquant.net/perseus/). Perseus was also used for hierarchical clustering of bradyzoite- and tachyzoite-specific surface antigens of the SRS family using RNAseq data of Hehl and colleagues [82], available on www.Toxodb.org.

### Co-immunoprecipitation and mass spectrometry identification

Parasites of the cKD HA-TgHCF101 transgenic cell line were treated for 3 days in the presence or absence of ATc in T175 cm^2^ seeded with Vero cells. After treatment, intracellular parasites were released by scraping of the host cells and 3 passages through a 26G needle. To eliminate cell host debris, parasites were filtered through fiber glass wool and harvested by centrifugation at 650 g for 5 min and washed 3 times in DPBS (Gibco). Parasites were resuspended in lysis buffer (1%NP40, 50 mM Tris-HCl (pH 8), 150 mM NaCl, 4 mM EDTA, supplemented with cOmplete Mini protease inhibitors mix (Roche)) and incubated overnight at 4°C on a rotating wheel. Centrifugation of insoluble material was performed at 13,500 g for 30 min at 4°C. The supernatant was transferred to a tube containing 50 μl of anti-HA magnetic beads (Thermo Fisher, 88836) for 4 h at 4°C on a rotating wheel. The depleted fraction was then removed and beads were washed 5 times with lysis buffer. For the elution of immunoprecipitated proteins, beads were incubated in 50 μl of HA peptide solution at 2 mg/ml (Thermo Fisher, 26184) for 1 h at 37°C on a rotating wheel. The eluted proteins were mixed with Laemmli buffer and resolved on a 10% SDS-Page for 35 min at 100V. In-gel proteins were fixed with a combination of acetic acid and ethanol and stained with PageBlue Protein Staining Solution (Thermo Scientific). Each lane was cut in 3 identical pieces which were digested with trypsin, peptide extraction was done as previously described [80] and mass spectrometry identification was performed as described for label-free quantitative proteomic.

Proteomic data were analyzed on R following the publicly available script of DEP analysis package (https://github.com/arnesmits/DEP, v.1.7.1). The following parameters were used for differential expression analysis: data set was normalized by variance-stabilizing transformation and missing values were imputed via MNAR (missing not at random) method, the fold change cutoff was set at 1.5 and *p*-value at 0.05. Statistical analyses were performed by a differential enrichment test based on protein-wise linear models and empirical Bayes statistics [83], and *p*-values were adjusted by the Benjamini–Hochberg correction.

Validation of co-immunoprecipitation mass spectrometry results was performed by immunoblotting. C-terminally c-myc tagged candidates in the cKD HA-TgHC101 background were grown in the presence or absence of ATc for 48 h. Parasites were harvested and subjected to co-immunoprecipitation using myc-Trap agarose beads (Chromotek, yta-20) following manufacturer’s instructions. Cross-linking of proteins was performed prior to co-immunoprecipitation for the cKD HA-TgHCF101 CIA1-myc cell line using 1% (w/v) PFA (diluted in PBS) for 5 min at room temperature and quenched with 125 mM ice-cold glycine. Immunoprecipitated proteins were then resolved on 10% SDS-Page prior to immunoblot analysis.

### Yeast two-hybrid

RNA was extracted from parasites using the NuceloSpin RNA kit (Macherey-Nagel). cDNA was obtained using SuperScript III First Strand Synthesis SuperMix for RT-qPCR (Invitrogen) using oligodT and following the manufacturer’s instructions. Specific cDNAs were subsequently amplified using KOD DNA polymerase (Novagen). Cloning was performed with In-Fusion HD cloning kit (Takara) for each candidate into pGADT7 and pGBKT7 vectors (Clontech, Takara Bio) to allow expression of AD-(Gal4 activation domain) and BD-(Gal4 DNA binding domain) fusion proteins, respectively. Primers were designed using the InFusion Cloning Primer Design Tool (https://takarabio.com/) and are listed in S5 Table.

All experiments were performed in the Gal4-based yeast two-hybrid (Y2H) reporter strain YRG2 (MATα, *ura3-52*, *his3-200*, *ade2-101*, *lys2-801*, *leu2-3*, *112*, *trp1-901*, *gal4-542*, *gal80-538*, *lys2*::UAS_GAL1_-TATA_GAL1_-HIS3, URA3::UAS_GAL4_,_*17MERS(x3)*_-TATA_CYC1_-LacZ) (Stratagene, Agilent). Yeast cells were co-transformed by pGAD/pGBK construct pairs and selected on plates containing the minimal YNB medium (0.7% Yeast Nitrogen Base medium without amino acids, 2% glucose, 2% Difco agar) supplemented with histidine (H), adenine (A), Lysine (K), and uracil (U) according to Gietz and Woods [84]. For all Y2H interaction assays, individual co-transformed colonies were cultured on liquid YNB+HUKA, adjusted to an OD_600_ of 0.05 and dotted on YNB+UKA plates containing (+His plates) or missing (-His plates) histidine (7 μl per dot). Plates were incubated at 30°C and cell growth was recorded after 5 days. Constructs pairs including empty vectors (AD-fusion + empty BD or empty AD + BD-fusion) were tested first to evaluate the capacity of each protein of interest to transactivate the *HIS3* reporter gene in the absence of interactor and to determine the appropriate 3-aminotriazole (3AT) concentration required to abolish false interactions. True positive interactions were visualized as cells growing on YNB+UKA in the absence of histidine (-his plates) and eventually in the presence of 3AT if required. Increasing 3AT concentrations were also used to challenge the strength of positive interactions. For all assays, at least 3 independent transformations per construct pair were performed.

### DNA content analysis

DNA content analysis by flow cytometry was performed as described previously [85]. Parasites from the cKD HA-TgHCF101 strain and from the background TATi ΔKu80 line were treated for up to 4 days in the presence or absence of ATc. Extracellular parasites were removed by washing with HBSS and intracellular parasites were released from host cells by scraping with a cell scraper (VWR, 734–2602), passages through 26G needles and finally filtered on a fiber glass wool column. Parasites were then fixed overnight in a solution of 70% ethanol and 30% PBS at 4°C. After fixation, parasites were washed in PBS and stained with a 30 µM propidium iodide solution for 30 min. Parasites DNA content was then analyzed by flow cytometry with an Aurora cytometer (Cytek) from the MRI facility. Raw FCS files and gating strategy for cytometry experiments are available at http://flowrepository.org/id/FR-FCM-Z8SR.

### Gliding motility assay

Parasites from the parental TATi ΔKu80 and the cKD HA-TgHCF101 transgenic cell lines were grown in the presence or absence of ATc for up to 4 days, and 1.10^6^ freshly egressed parasites were harvested and resuspended in 400 μl motility buffer (155 mM NaCl, 3 mM KCl, 2 mM CaCl_2_, 1 mM MgCl_2_, 3 mM NaH_2_PO_4_, 10 mM HEPES, 10 mM glucose) and 100 μl of the suspension was immediately placed on poly-L-lysine coated slides and incubated at 37°C for 15 min. Unattached parasites were removed by performing 3 washes with PBS. Finally, attached parasites were fixed with 4% (w/v) PFA and stained with anti-SAG1 antibody. Trails length (at least 100 per condition) were captured on random fields with a 63× objective on a Zeiss AXIO Imager Z2 epifluorescence microscope and were quantified using the NeuronJ plug-in on the ImageJ software as described previously [86].

### Rhoptry secretion assay

Rhoptry secretion was quantified by performing an evacuole assay [87]. Parasites from the cKD HA-TgHCF101 strain and cKD TgARO strain were grown in the presence or absence of ATc for up to 72 h. Freshly egressed parasites were then pretreated for 10 min in DMEM supplemented with 1 μm cytochalasin D (cytD), an inhibitor of actin polymerization. Parasites were allowed to secrete their rhoptry content as they were put in contact with HFF cells for 15 min in the presence of cytD at 37°C. Parasites and HFF cells were then immediately fixed with 8% (w/v, in PBS) PFA, permeabilized with 0.1% Triton X-100 and stained with anti-ROP1 and anti-SAG1 to detect secreted rhoptry material and parasites, respectively. For each technical replicate, 20 random fields were quantified and 3 technical replicates were performed for each of the 3 biological replicates.

### Statistical analyses

Statistical analyses were generally performed with the Prism 8.3 software (Graphpad). For proteomics experiments, statistics were performed with version 4.2.1 of the R package (2022-06-23, https://www.R-project.org/) using the Differential Enrichment analysis of Proteomics Data (DEP, v.1.23.0) package. Unless specified, values are expressed as means ± standard deviation (SD).

## Supporting information

S1 Fig Phylogenetic analysis of selected HCF101 homologs. Unrooted tree was generated by aligning sequences from HCF101 homologs, and the resulting alignment was submitted to phylogenetic analysis with the maximum likelihood method. Bootstrap values, inferred from 500 replicates, are indicated at the nodes. Scale bar represents 0.5 residue substitution per site. The data underlying this figure can be found in https://doi.org/10.6084/m9.figshare.28238195.
(TIF)

S2 Fig TgHCF101 is not associated with the apicoplast or its metabolism. **(A)** Fluorescent imaging of *T*. *gondii* tachyzoites ectopically expressing a GFP-fused copy of TgHCF101, DNA was stained with DAPI. Scale bar = 5 μm. **(B)** Immunoblot analysis of the lipoylation profile of *T*. *gondii* tachyzoites, typically showing apicoplast (Pyruvate dehydrogenase subunit E2, PDH-E2) and mitochondrial (Branched-chain 2-oxo acid dehydrogenase, BCDH-E2 and 2-oxoglutarate dehydrogenase, OGDH-E2) proteins that are largely unaffected upon TgHCF101 depletion by incubation with ATc for up to 4 days. Anti-SAG1 antibody is used as a loading control.
(TIF)

S3 Fig TgHCF101 depletion does not initially impact parasite motility. **(A)** Representative images of a gliding motility assay selected out of 3 independent replicates. The trails left behind by gliding parasites were detected using an anti-SAG1 antibody. TgHCF101 conditional mutant and parental cell line (TATi ΔKu80) were grown for 3 or 4 days in the presence of ATc. **(B)** Quantification of SAG1 trail length showed in (A) produced by parasites measured on 10 randomly selected fields. At least 100 trails were measured for each data set, values represented are means of *n* = 3 independent biological replicates (red and blue circles represent data points from these replicates), **** *p*-value ≤0.0001, Student’s *t* test. The data underlying this figure can be found in S1 Data.
(TIF)

S4 Fig Additional examples of morphological defects caused by TgHCF101 depletion. Electron microscopy was performed on cKD-TgHCF101 parasites pre-incubated with ATc for 24 h before being released from their host cell and allowed to reinvade for 24 h in the presence of ATc (+ATc). CC: cytoplasmic cleft, D: daughter bud, LD: lipid droplet, M: mitochondrion, N: nucleus, V: vacuole; ‘ and “denote magnifications of different regions of the same lettered image.
(TIF)

S5 Fig Immunofluorescence analysis of cKD HA-TgHCF101 parasites incubated or not with ATc for 4 days, showing organellar defects upon TgHCF101 depletion. Mito: mitochondrion, labeled with an anti-F1β ATPAse antibody; Apico: apicoplast, labeled with an anti-PDH-E2 antibody; IMC: inner membrane complex, labeled with an anti-IMC3 antibody; PM: plasma membrane, labeled with an anti-SAG1 antibody; DNA was labeled with DAPI. Scale bar = 5 μm.
(TIF)

S6 Fig Lipid droplet induction depends on TgHCF101 depletion and is not responsible for parasite demise. **(A, B)** Correspond to quantification of the number and area of lipid droplets, respectively, in apicoplast (cKD-TgSUFC) and mitochondrion (cKD-TgISU1) Fe-S cluster synthesis mutants grown in the absence or presence of ATc for up to 72 h; 100 parasites were analyzed per condition. Values are the mean ± SD of *n* = 3 independent biological replicates; ns, not significant (*p*-value ≥0.05, Student’s *t* test). **(C)** Immunofluorescence assay of parasites from the cKD-TgHCF101 cell line treated for 72 h with or in the absence of ATc and supplemented or not with diacylglycerol acyltransferase inhibitor T863. Lipid droplets were detected with Nile red (orange), parasites are outlined with anti-IMC3 antibody (green), and DNA is stained with DAPI. Scale bar = 5 μm. For this experiment and the following analyses involving T863, the drug was used at 5 μm and an equivalent volume of the vehicle only (DMSO) was used in the control conditions. **(D, E)** Correspond to the quantification of the number and area of lipid droplet, 100 parasites were analyzed per condition. Parasites were grown in the absence of ATc for the parental (TATi ΔKu80) and transgenic (cKD HA-TgHCF101) cell lines or for 72 h with ATc treatment, supplemented or not with T863. Values are represented as the mean ± SD of *n* = 3 independent biological replicates; ns, not significant (*p*-value ≥0.05), * *p*-value ≤0.05, ** *p*-value ≤0.01, and *** *p*-value <0.001, Student’s *t* test. **(F)** Plaque assays were carried out by infecting a monolayer of HFFs with cKD HA-TgHCF101 cell lines for 7 days in the presence or absence of ATc and supplemented or not with T863. The data underlying this figure can be found in S2 and S3 Table and in S1 Data.
(TIF)

S7 Fig Generation of a TgNAR1 conditional mutant. **(A)** Strategy for generating the inducible knockdown of *TgNAR1* by promoter replacement in the TATi ΔKu80 cell line. **(B)** Diagnostic PCR for checking correct integration of using the primers mentioned in (**A**), on genomic DNAs of a transgenic parasite clone and of the parental strain. **(C)** Semi-quantitative RT-PCR was used to verify the efficient down-regulation of *TgNAR1* upon addition of ATc. Reverse transcriptase was omitted in the “no RT” control. *β-tubulin* was used as a control. **(D)** Plaque assay revealed that depletion of TgNAR1 is important for parasite growth.
(TIF)

S8 Fig Depletion of TgHCF101 has no extensive impact on rhoptries. **(A)** Volcano plot showing differential expression of proteins impacted by TgHCF101 depletion after 72 h of ATc treatment analyzed by label-free quantitative proteomic. X-axis correspond to the log_2_ of the Fold-change, Y-axis correspond to the -log_10_ of the p-value when comparing cKD-TgHCF101 expression values to the TATi ΔKu80 parental cell line. Statistical analyses were performed with ANOVA from 4 independent biological replicates. Cut-offs were set at ≤1.5- or ≥1.5-FC and *p*-value ≤0.05. Significant hits corresponding to rhoptry bulb protein proteins were highlighted in purple on the graph. **(B)** Immunofluorescence assay of parasites from the cKD HA-TgHCF101 cell line pretreated for 48 h and allowed to grow on HFF-coated coverslips for another 24 h with or in the absence of ATc. Rhoptries were detected with anti-ARO antibody (green), parasites are outlined with white dotted lines and DNA is stained with DAPI. Scale bar = 5 μm**. (C)** Quantification of rhoptry secretion events (evacuoles) in the cKD HA-TgHCF101 mutant upon TgHCF101 depletion for 72 h. The TgARO conditional knock-down cell line serves as a control of rhoptry secretion defect upon ATc treatment. Parasites with and without evacuoles were counted on 20 randomly selected fields, with 3 technical replicates for each biological replicate. Values are represented as the mean ± SD of *n* = 3 independent biological replicates; ns, not significant (*p*-value ≥0.05), * *p*-value ≤0.05, **** a *p*-value ≤0.001, Student’s *t* test. **(D)** Immunoblot analysis of the expression of protein TgROP7 upon depletion of TgHCF101 for 3 days. The pro-form of TgROP7 (pROP7) is highlighted by a red arrow and the mature form (mROP7) by a blue arrow. TgROP7 signal was detected by anti-ROP7 antibody and anti-actin antibody was used as a loading control. The data underlying this figure can be found in S1 Data.
(TIF)

S9 Fig Constructs for tagging TgABCE1 and TgCIA1 in the cKD HA-TgHCF101 mutant background. **(A, C)** Correspond to schematic representations of the strategy used to add a C-terminal myc tag to proteins TgABCE1 (**A**) and TgCIA1 (**C**) by homologous recombination at the native locus. Chloramphenicol was used to select transgenic parasites. **(B, D)** Correspond to diagnostic PCRs on genomic DNA from parental cell line (cKD HA-TgHCF101) or new clonal cell lines, in order to check for the integration of the sequence coding for the myc tag using primers highlighted on the (**A**) and (**C**) schemes.
(TIF)

S10 Fig Generating a conditionally TgHCF101 mutant in the TgPOLD1-HA cell line. **(A)** Strategy for generating the inducible knockdown of *TgHCF101* by promoter replacement in the TgPOLD1-HA cell line. **(B)** Diagnostic PCR for checking correct integration of using the primers mentioned in (**A**), on genomic DNAs of a transgenic parasite clone and of the parental strain. **(C)** Semi-quantitative RT-PCR was used to verify the efficient down-regulation of *TgHCF101* upon addition of ATc. Reverse transcriptase was omitted in the “no RT” control. *β-tubulin* was used as a control. **(D)** Plaque assay confirmed that depletion of TgHCF101 is important for parasite growth. **(E)** Nile red staining confirmed that depletion of TgHCF101 in the cKD TgHCF101 TgPOLD1-HA cell line induces lipid droplet formation.
(TIF)

S11 Fig No direct interaction between TgHCF101 and TgNTHL1 was detected in a Gal4-based yeast two-hybrid assay. Co-transformed YRG2 cells expressing AD- and BD-fusion proteins were plated on a control plate (+His, upper panel) for checking cell fitness and on the Y2H test plate (-His, mid panel), and plates were incubated at 30°C. Yeast growth was recorder after 5 days. At the cell concentration used (OD_600_ = 0.05), the observed interaction between AD-TgHCF101 and BD-TgABCE1 fusion proteins was strong enough to allow cell growth in the presence of 5 mM 3AT inhibitor (-his + 3AT lower panel) but no interaction was detected with TgNTHL1. None of the proteins tested alone exhibited HIS3 transactivation capacities. Results shown here are representative of 3 independent experiments.
(TIF)

S12 Fig CIA adapters YAE1 and LTO1 are restricted to specific eukaryotic lineages. Sunburst representation of the distribution of YAE1 and LTO1 domains as retrieved in the Interpro database (https://www.ebi.ac.uk/interpro/).
(TIF)

S1 Table Sequences used for the phylogenetic analysis displayed in Fig 1A. Abbreviations used in the Fig 1A phylogenetic analysis are mentioned in red next to each sequence.
(XLSX)

S2 Table Proteins with higher expression upon depletion of TgHCF101 as found by label-free quantitative proteomics. For each protein candidate (with www.ToxoDB.org and www.Uniprot.org identifier), log_2_ of the different ratio were calculated between the mean MaxQuant LFQ values found for the cKD HA-TgHCF101 mutant and the TATi ΔKu80 parental cell line. -log_10_(pvalue) is also provided. Putative subcellular localization was obtained from the hyperLOPIT data available on ToxoDB.org or by manual annotation. CRISPR fitness score and transcriptomic data for tachyzoites (Tz) and bradyzoites (Bz) were obtained from ToxoDB.org.
(XLSX)

S3 Table Proteins with lower expression upon depletion of TgHCF101 as found by label-free quantitative proteomics. For each protein candidate (with www.ToxoDB.org and www.Uniprot.org identifier), log_2_ of the different ratio were calculated between the mean MaxQuant LFQ values found for the cKD HA-TgHCF101 mutant and the TATi ΔKu80 parental cell line. -log_10_(pvalue) is also provided. Putative subcellular localization was obtained from the hyperLOPIT data available on ToxoDB.org or by manual annotation. CRISPR fitness score and transcriptomic data for tachyzoites (Tz) and bradyzoites (Bz) were obtained from ToxoDB.org.
(XLSX)

S4 Table Proteins identified as co-immunoprecipitating with TgHCF101 through comparative mass spectrometry analysis of immunoprecipitated extracts form the cKD HA-TgHCF101 cell line grown or not in the presence of ATc. Proteins enriched (TgHCF101-expressing sample vs. TgHCF101-depleted control) in a statistically significant way (*p*-value ≤0.05 and Benjamini–Hochberg correction) are in green (dark green for TgHCF101 and putative iron–sulfur protein) and those less abundant are in red.
(XLSX)

S5 Table Oligonucleotides used in this study. (XLSX)

S1 Raw images Raw images for immunoblots and gels in Figs 1D, 1E, 4C, 4D, 6C, 6F, 7B, 7C, 7E, S2B, S7B, S7C, S8D, S9B, S9D, S10B, and S10C. (PDF)

S1 Data Numerical Data for Figs 2B, 2D, 3C, 3D, 4H, 4I, 5D, 6D, 6G, 7F, S3B, S6A, S6B, S6D, S6E, and S8C. (XLSX)

## Abbreviations

ATc: anhydrotetracycline
BSA: bovine serum albumin
CAT: chloramphenicol acetyltransferase
cKD: conditional knock-down
CIA: cytosolic iron–sulfur cluster assembly
CTC: CIA targeting complex
DAPI: 4′,6-diamidino-2-phenylindole
DHFR-TS: dihydrofolate reductase thymidylate synthase
DIC: differential interference contrast
DMEM: Dulbecco’s Modified Eagle Medium
FBS: fetal bovine serum
HA: hemagglutinin
HBSS: Hank’s balanced salt solution
HCF101: high chlorophyll fluorescence 101
HFF: human foreskin fibroblast
IFA: immunofluorescence assay
IMC: inner membrane complex
ISC: iron sulfur cluster
LD: lipid droplet
MNAR: missing not at random
ns: not significant
PBS: phosphate-buffered saline
PDH-E2: E2 subunit of pyruvate dehydrogenase
PFA: paraformaldehyde
ROS: reactive oxygen species
SD: standard deviation
SUF: sulfur mobilization
TCR: targeting complex recognition
