# Supplementary material for: The HCF101 protein is an important component of the cytosolic iron–sulfur synthesis pathway in Toxoplasma gondii
Source: PLoS Biol. 2025 Feb 6;23(2):e3003028. doi: 10.1371/journal.pbio.3003028 (PMC11838916; doi:10.1371/journal.pbio.3003028)

Original images for gels and blots

Fig. 1D

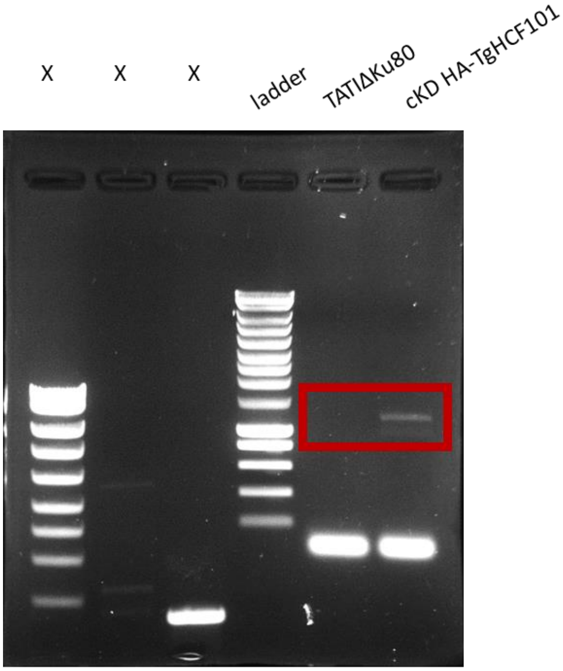

Fig 1.D

Fig. 1E

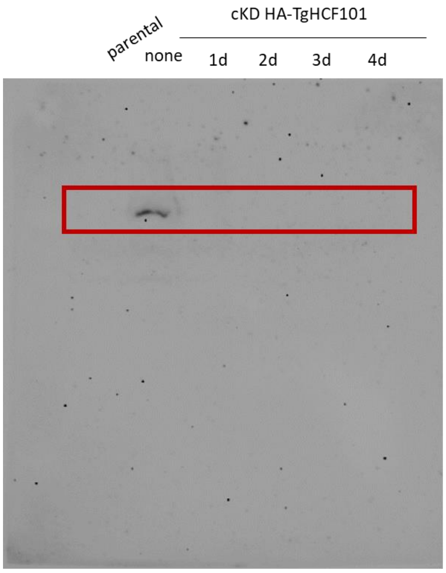

Fig 1.E
anti-HA

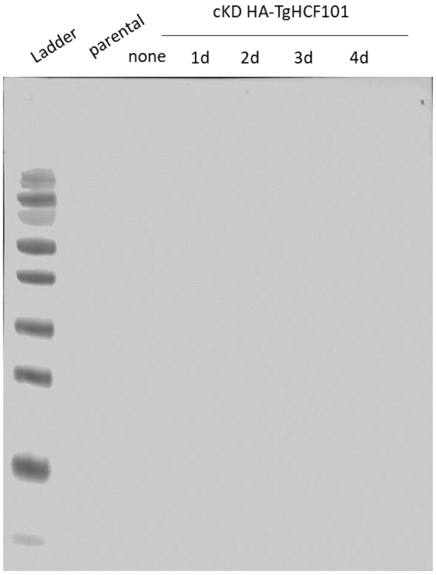

Fig 1.E
colorimetric

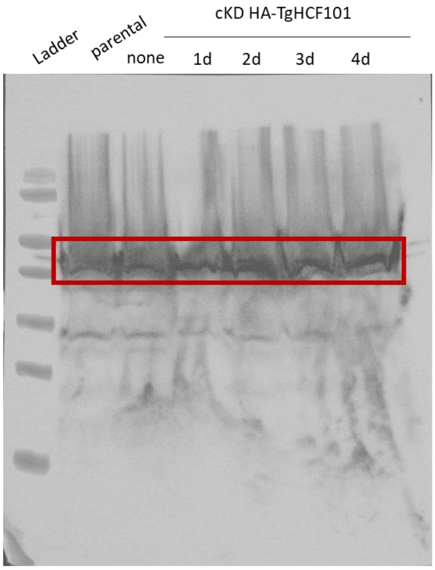

Fig 1.E anti-actin
HRP + colorimetric
(composite)

Fig. 4C

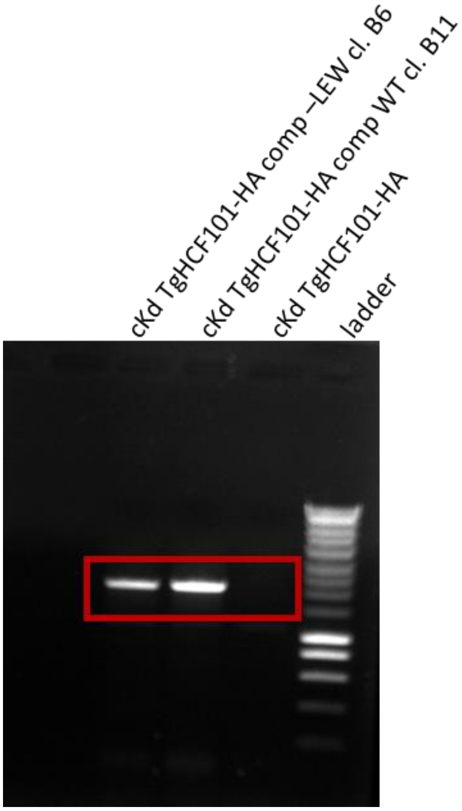
cKd TgHCF101-HA comp –LEW cl. B6
cKd TgHCF101-HA comp WT cl. B11
cKd TgHCF101-HA
ladder

Fig. 4D

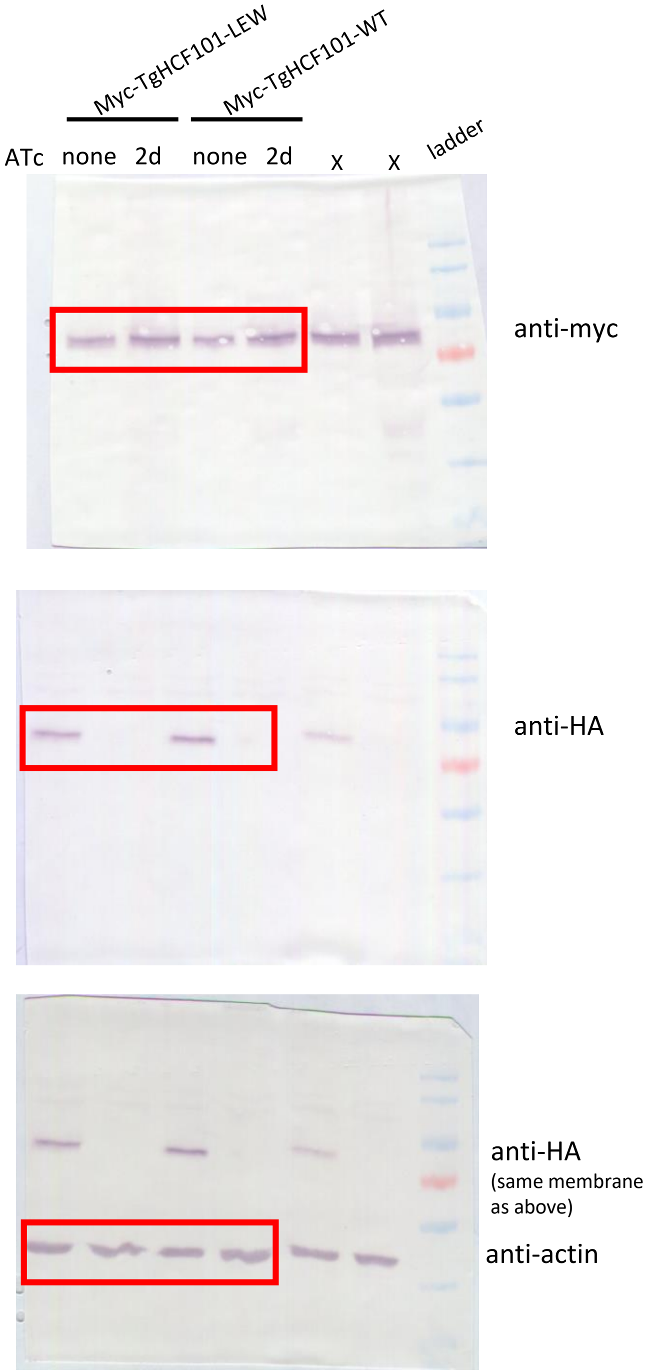
Myc-TgHCF101-LEW
Myc-TgHCF101-WT
ATc
none
2d
none
2d
x
x
ladder
anti-myc
anti-HA
anti-HA
(same membrane as above)
anti-actin

Fig. 6C

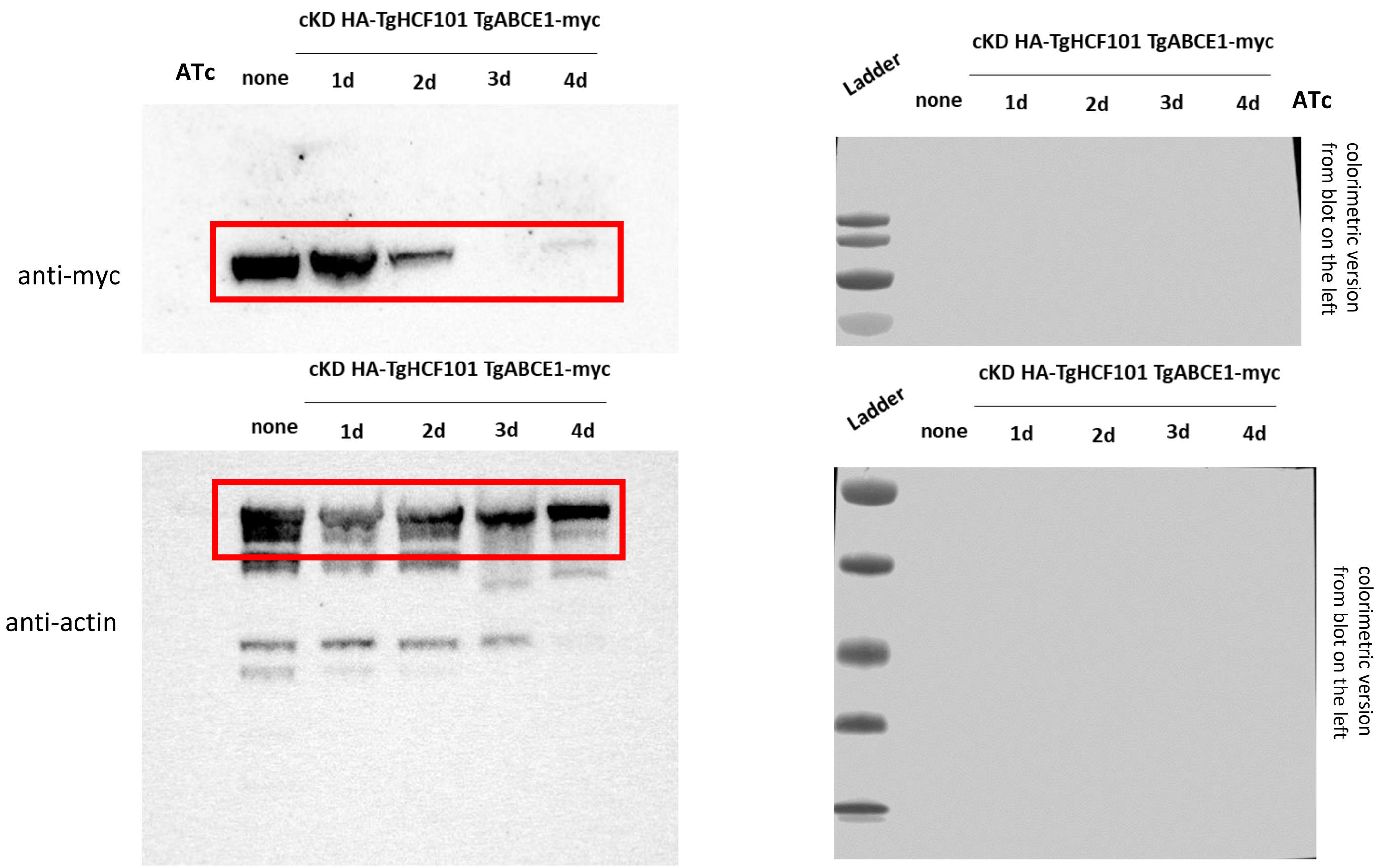
cKD HA-TgHCF101 TgABCE1-myc
ATc
none
1d
2d
3d
4d
anti-myc
anti-actin
Ladder
colorimetric version from blot on the left

Fig. 6F

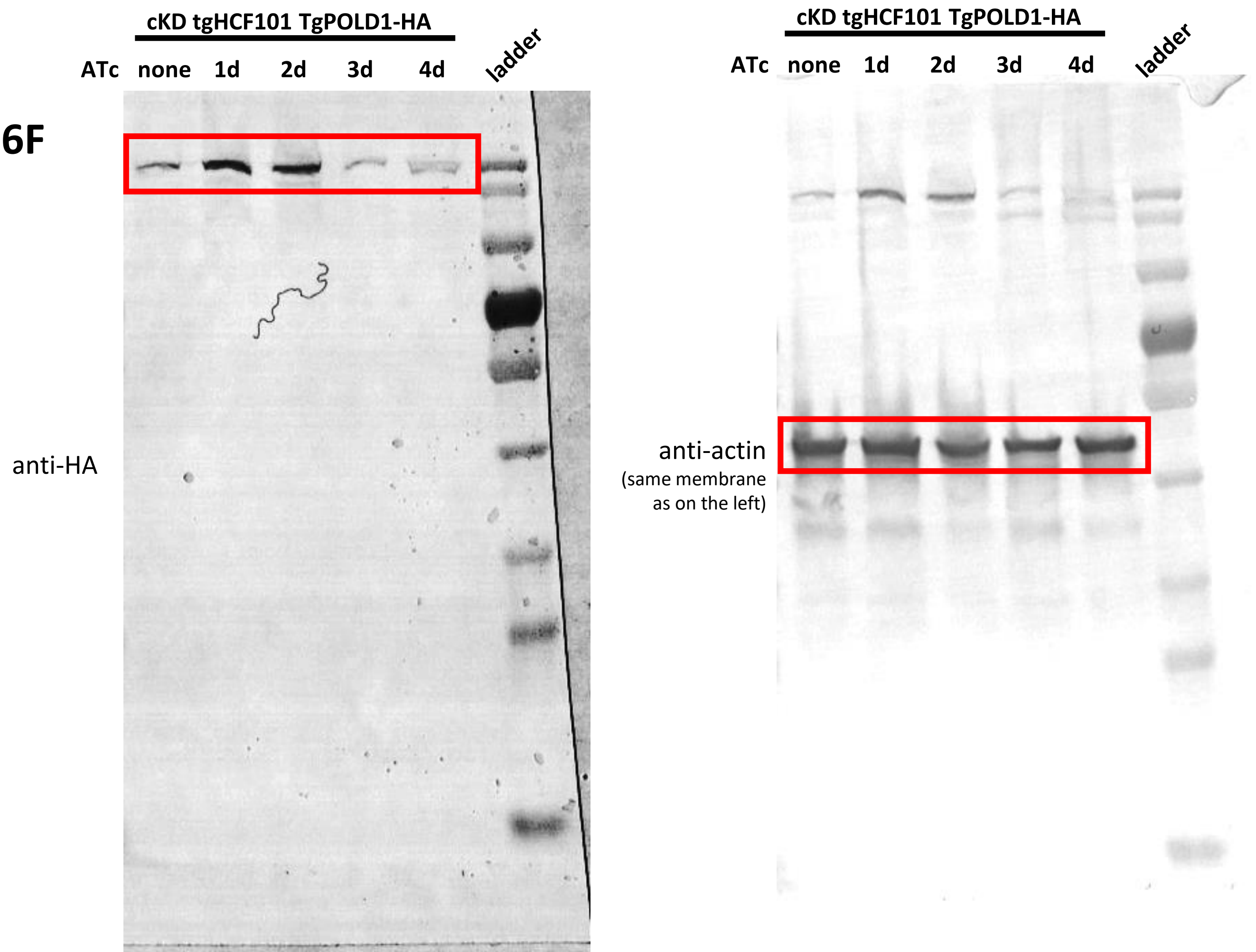
cKD tgHCF101 TgPOLD1-HA
ATc
none
1d
2d
3d
4d
ladder
anti-HA
anti-actin
(same membrane as on the left)

Fig. 7B

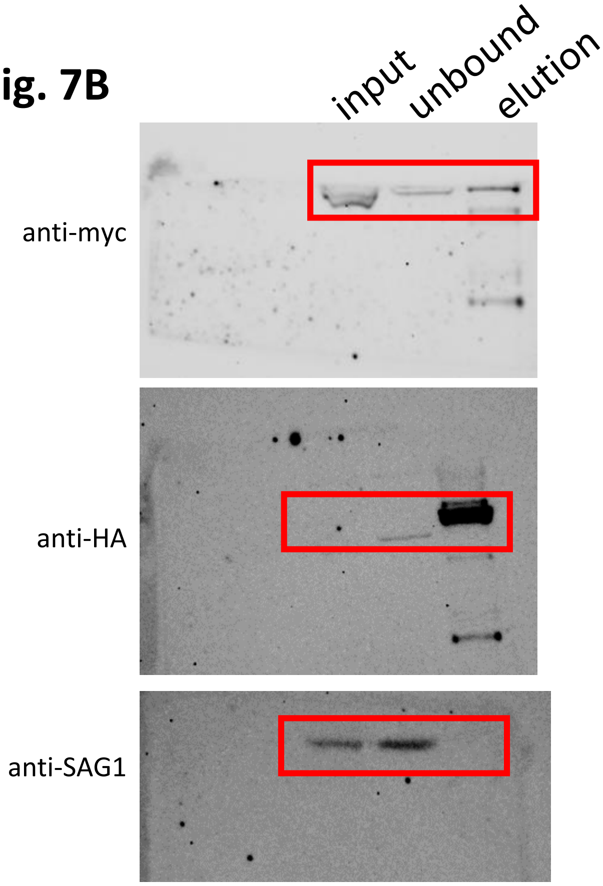
input
unbound
elution
anti-myc
anti-HA
anti-SAG1

Fig. 7C

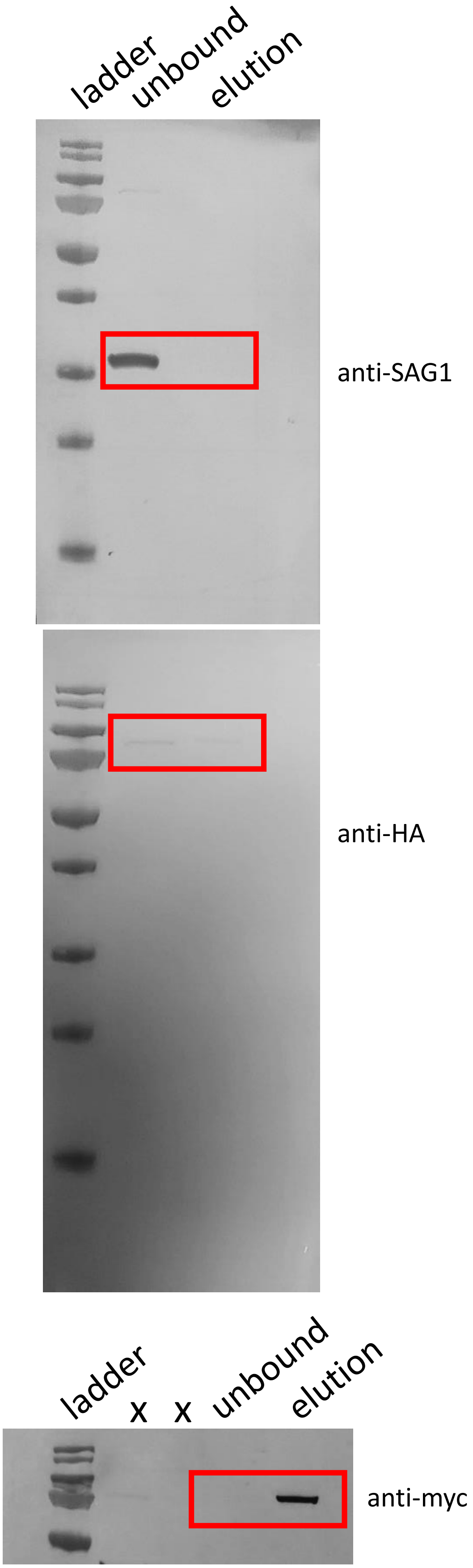
ladder
unbound
elution
anti-SAG1
anti-HA
ladder
x
x
unbound
elution
anti-myc

Fig. 7E

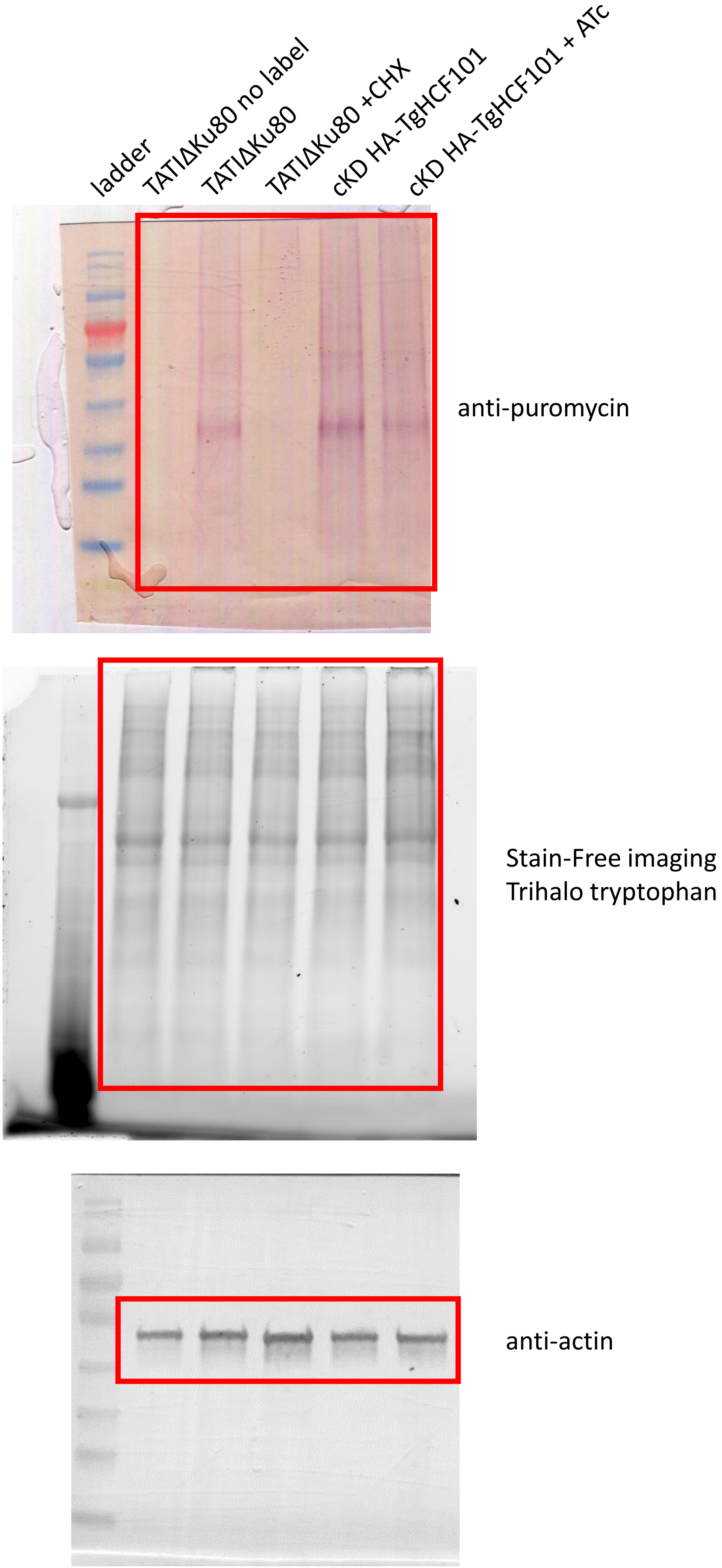
ladder
TATIΔKu80 no label
TATIΔKu80
TATIΔKu80 +CHX
cKD HA-TgHCF101
cKD HA-TgHCF101 + ATc
anti-puromycin
Stain-Free imaging
Trihalo tryptophan
anti-actin

S2B Fig.

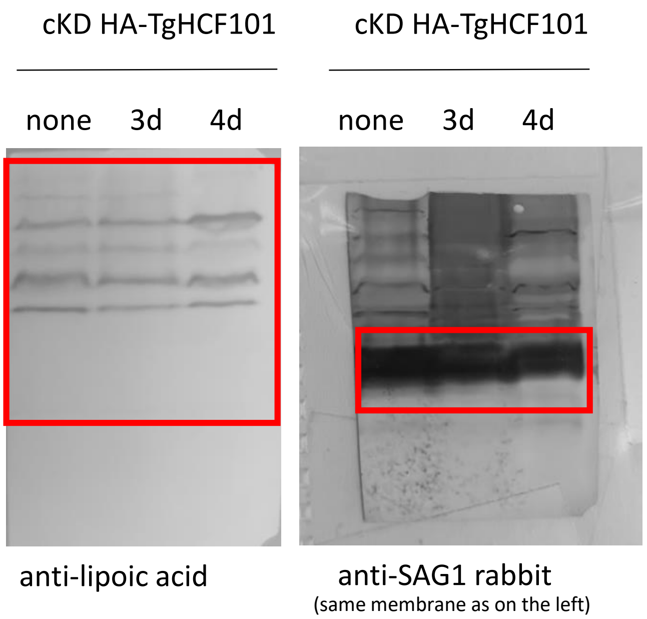

cKD HA-TgHCF101
none
3d
4d
anti-lipoic acid
cKD HA-TgHCF101
none
3d
4d
anti-SAG1 rabbit
(same membrane as on the left)

S7B Fig.

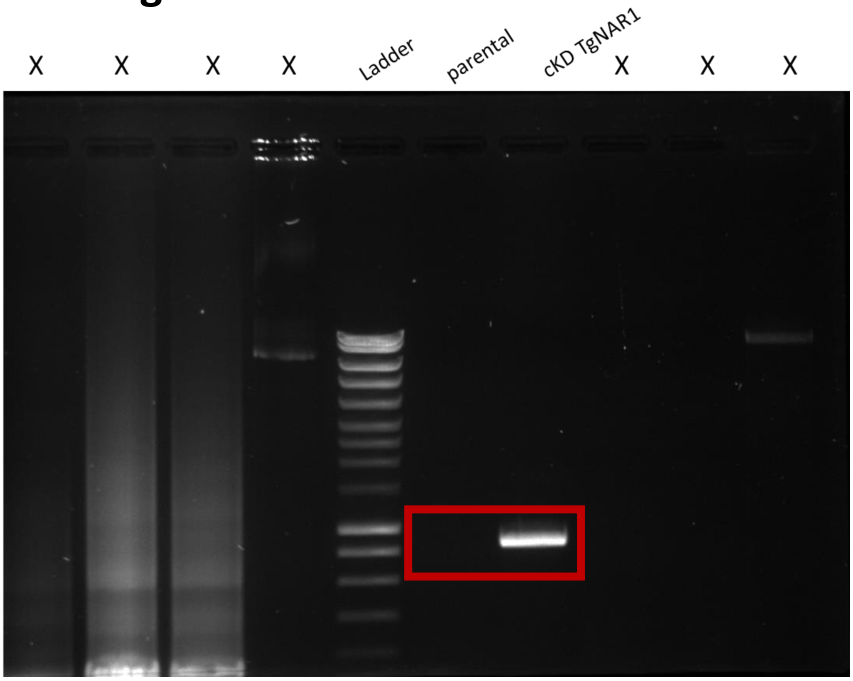

S7C Fig.

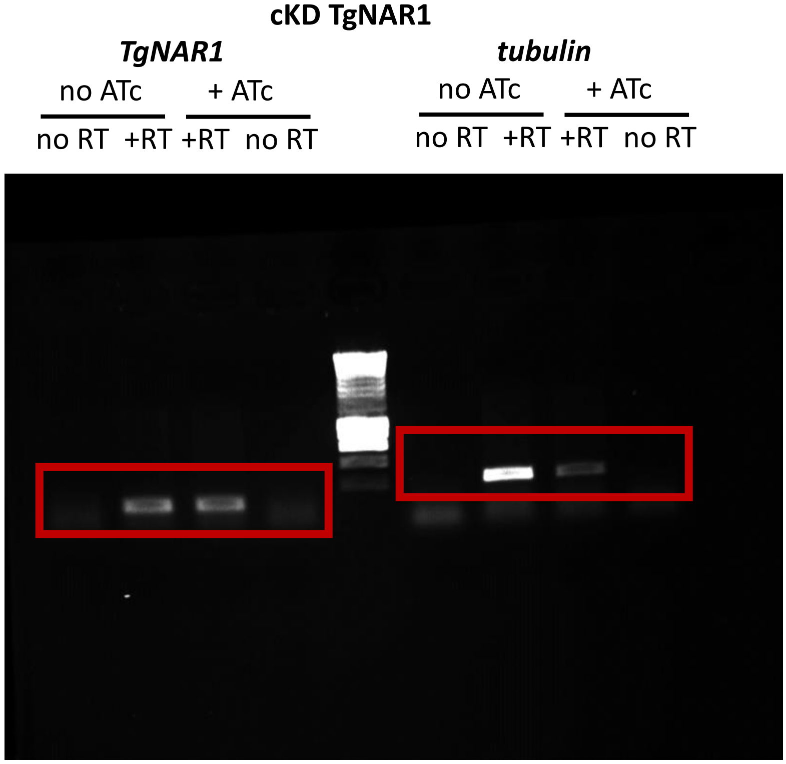

S8D Fig.

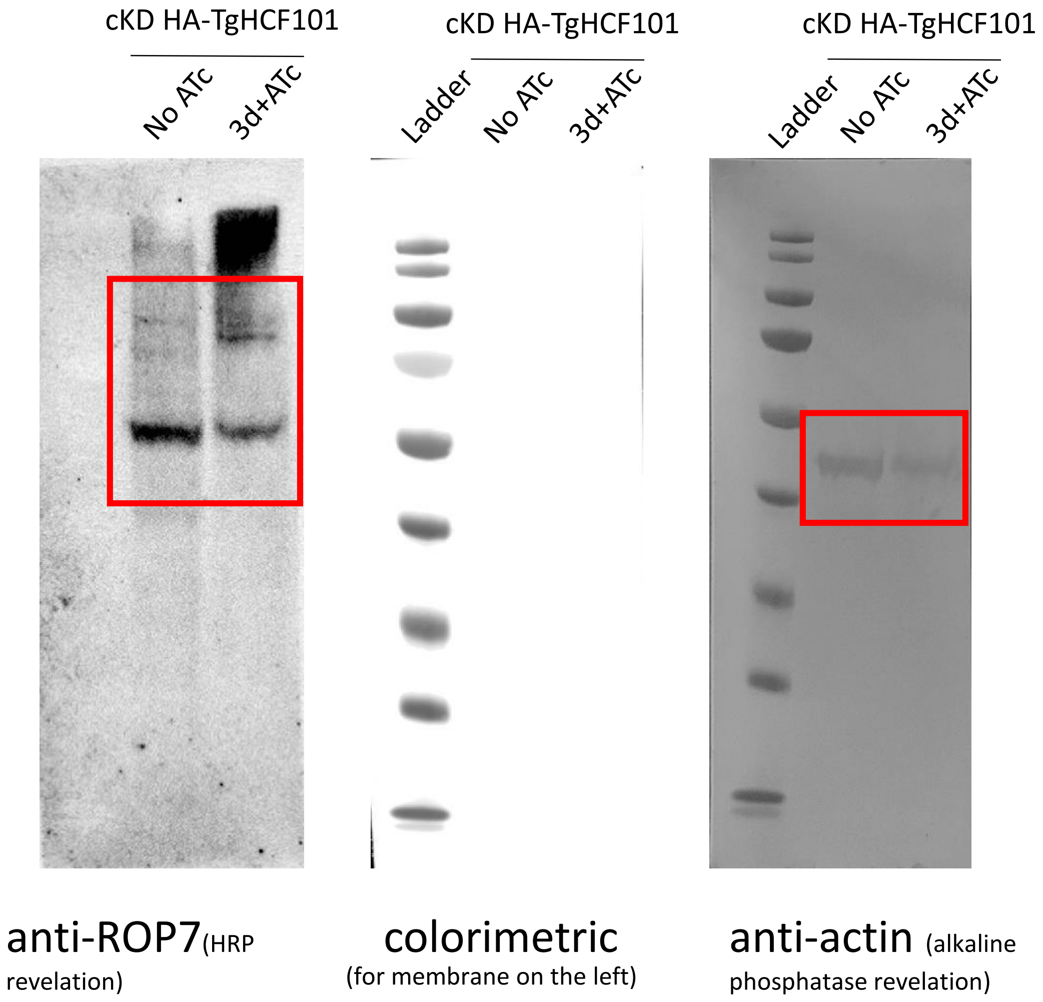
cKD HA-TgHCF101
No ATc
3d+ATc
cKD HA-TgHCF101
Ladder
No ATc
3d+ATc
cKD HA-TgHCF101
Ladder
No ATc
3d+ATc
anti-ROP7(HRP revelation)
colorimetric
(for membrane on the left)
anti-actin (alkaline phosphatase revelation)

S9B and D Fig.

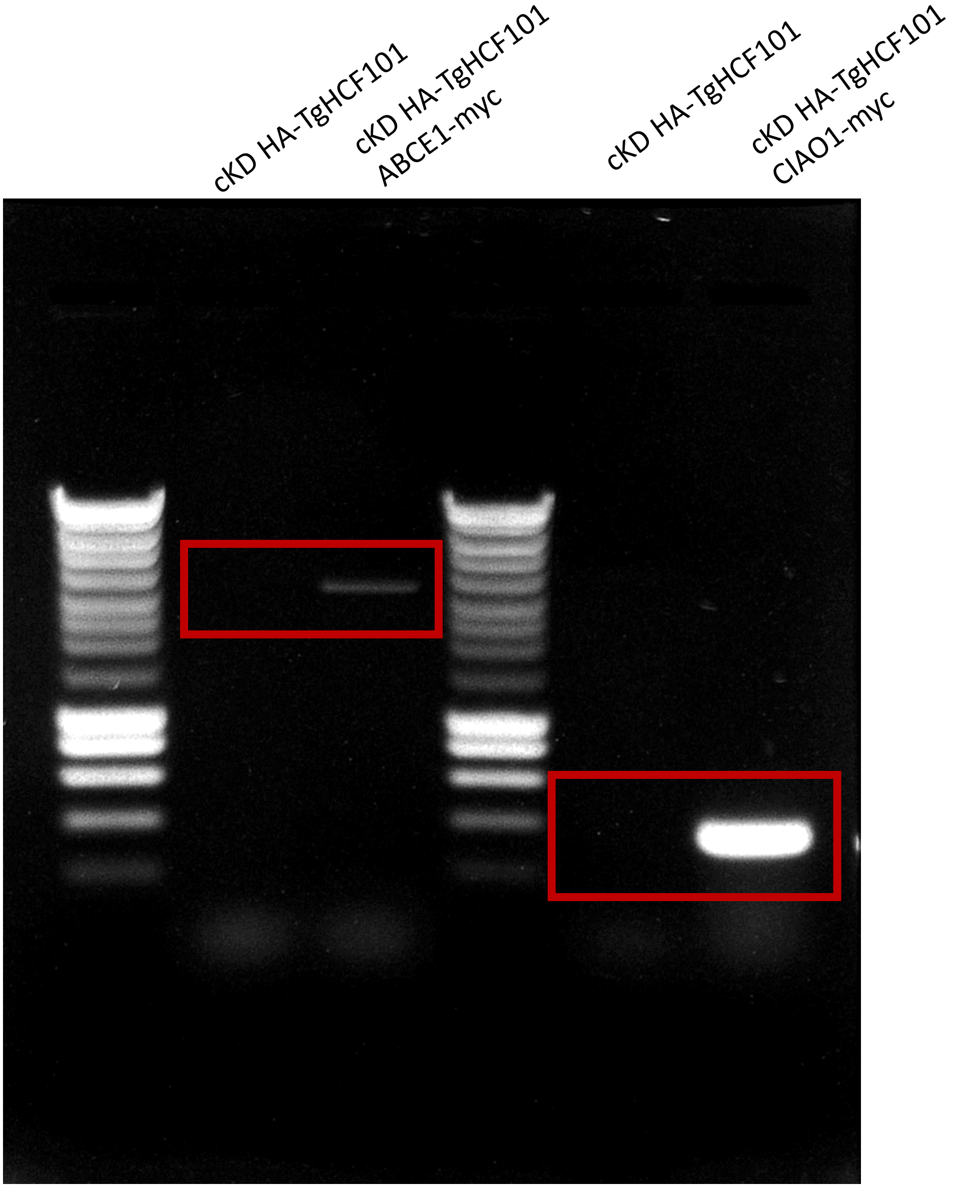
cKD HA-TgHCF101
cKD HA-TgHCF101 ABCE1-myc
cKD HA-TgHCF101
cKD HA-TgHCF101 CIAO1-myc

S10B Fig.

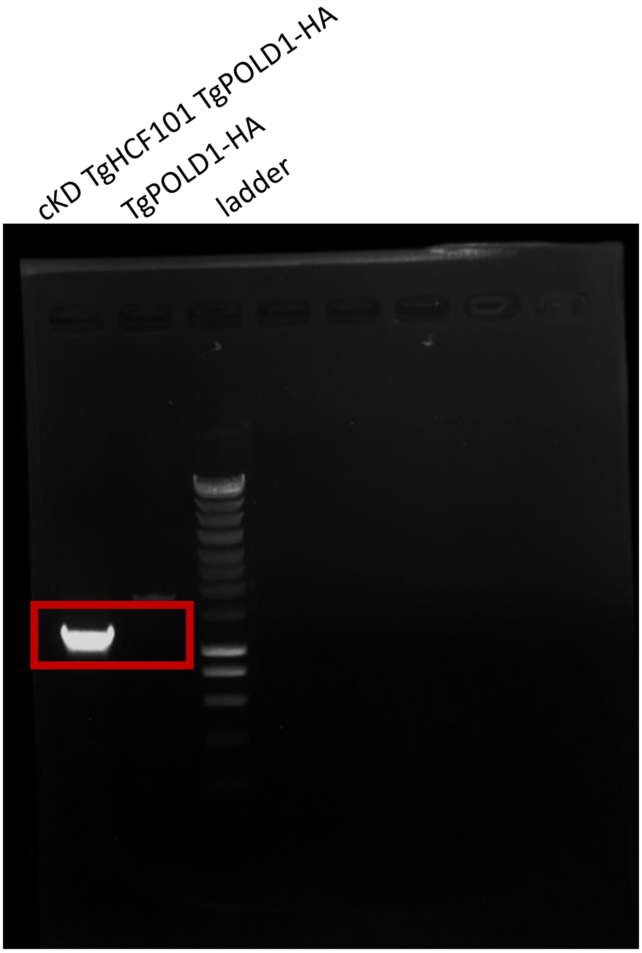

S10C Fig.

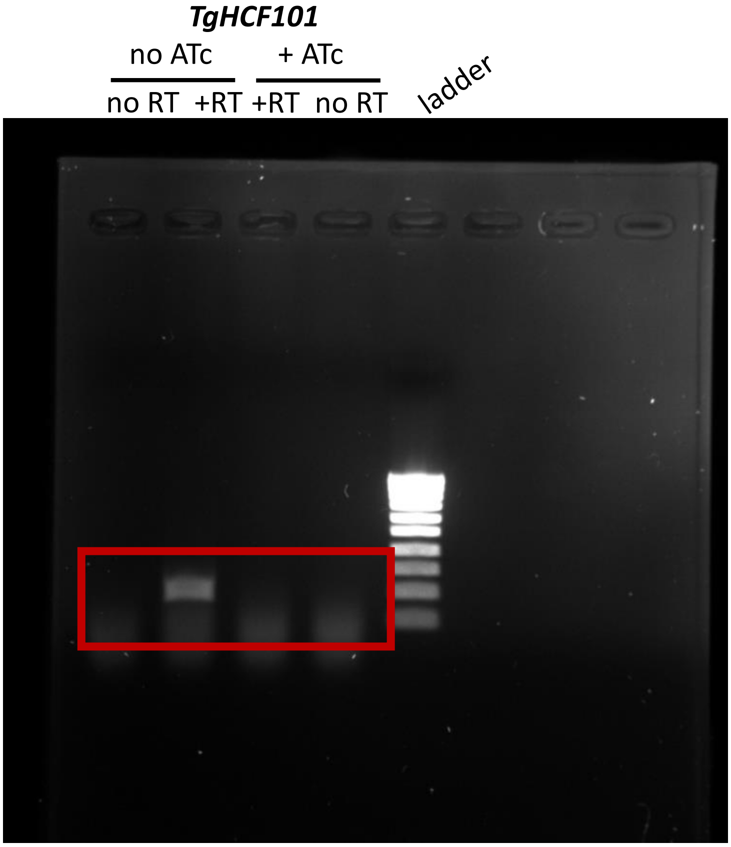

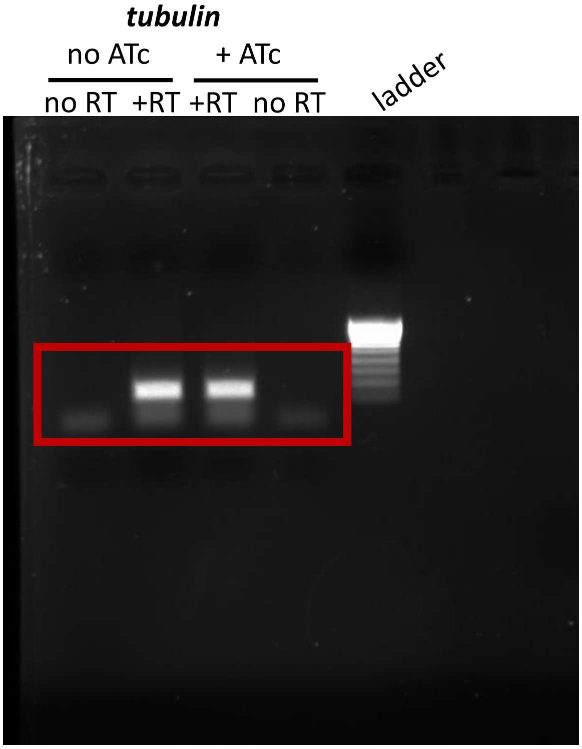

Supplement: S1 Raw images — (PDF) [file pbio.3003028.s018.pdf]
